# Sevoflurane Inhibits Layer 5 Pyramidal Neurons via Kv1.2‐Dependent Modulation of Subthreshold Currents

**DOI:** 10.1111/jnc.70360

**Published:** 2026-01-20

**Authors:** Aelton S. Araujo, Gabriel M. de Queiroz, Sérgio Ruschi B. Silva, Werner Treptow, Katarina E. Leao

**Affiliations:** ^1^ Hearing and Neuronal Activity Lab, Brain Institute Federal University of Rio Grande do Norte Natal Brazil; ^2^ Laboratório de Biologia Teórica e Computacional, Departamento de Biologia Celular Universidade de Brasília Brasília Brazil

**Keywords:** Kv1.2, layer 5, primary auditory cortex, pyramidal neurons, Sevoflurane, voltage‐gated potassium channels

## Abstract

General anesthetics reduce cortical activity and disrupt consciousness, yet the molecular mechanisms underlying their effects on neocortical neurons remain incompletely understood. Recent evidence implicates layer 5 pyramidal neurons (L5 PNs) as critical targets, particularly through anesthetic‐induced decoupling of distal apical dendritic inputs from somatic output. While several anesthetics impair L5 excitability, the ion channels mediating this effect have yet to be clearly identified. Voltage‐gated Kv1.2 potassium channels have emerged as compelling candidates due to their high expression in L5 PNs and their known potentiation by volatile anesthetics. In this study, we investigated the effects of low‐dose sevoflurane (~22 μM) on L5 PNs in the primary auditory cortex of adult mice using whole‐cell patch‐clamp recordings. Sevoflurane significantly suppressed firing and induced cell‐type‐specific changes in membrane properties: depolarizing the resting potential in type A neurons and increasing input resistance and altering action potential shape in type B neurons. Application of the selective Kv1.2 blocker TsTX‐Kα partially reversed these effects at subthreshold membrane potentials, implicating Kv1.2 channel potentiation in the modulation of neuronal excitability. Supporting that view, NEURON simulations using a detailed biophysical model of thick‐tufted L5b pyramidal neurons further revealed a significant sevoflurane‐induced increase in persistent K^+^ conductance, consistent with Kv1.2 potentiation. To our knowledge, this is the first study to demonstrate distinct, cell‐type‐specific effects of sevoflurane on L5 PNs and to establish the functional relevance of Kv1.2 channel potentiation in anesthetic suppression of cortical excitability. These findings offer new insights into the molecular actions of sevoflurane and support a broader role for Kv1.2 channels in mediating anesthetic‐induced outcomes.

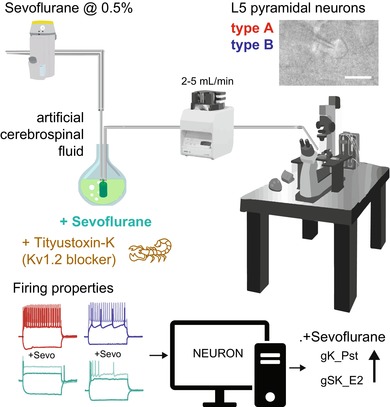

## Graphical abstract

General anesthetics suppress cortical activity, but their cellular mechanisms remain unclear. We investigated how the volatile anesthetic sevoflurane affects layer 5 pyramidal neurons in the auditory cortex. Using patch‐clamp recordings and computational modeling, we found that low‐dose sevoflurane reduces neuronal firing through Kv1.2 channel potentiation, which enhances subthreshold potassium currents and decreases excitability. This is the first demonstration of a Kv1.2‐dependent mechanism underlying anesthetic suppression of cortical neurons, providing new insight into how volatile anesthetics disrupt cortical processing and contribute to the loss of consciousness.

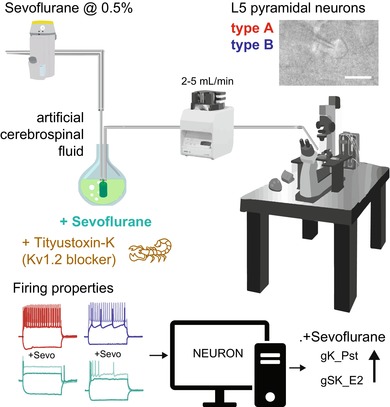

**Abbreviations**
aCSF: artificial cerebrospinal fluid
ADP: afterdepolarization
AHP: afterhyperpolarization
FDR: false discovery rate
HCN: hyperpolarization‐activated cyclic nucleotide‐gated channels
I_*h*_
: *h*‐current
ISI: inter‐spike interval
K_*2P*_
: tandem‐pore potassium channels
Kv: voltage‐gated potassium channel
L: layer
PN: pyramidal neuron
PTFE: polytetrafluoroethylene
TsTX‐Kα: tityustoxin‐Κα

## 1 Introduction

General anesthetics, including both volatile and intravenous agents, are chemically diverse small molecules—such as haloalkanes, haloethers, and alkylphenols. Despite over a century of research, their precise mechanisms of action at the cellular and molecular levels remain incompletely understood. Recent studies have increasingly emphasized the effects of general anesthetics on neocortical neurons, particularly layer 5 pyramidal neurons (L5 PNs), which exhibit synchronized firing patterns associated with critical anesthetic endpoints such as loss and recovery of consciousness (Suzuki and Larkum 2020; Bharioke et al. 2022).

Notably, agents such as ketamine, isoflurane, and urethane have been shown to reduce the excitability of L5 PN distal apical dendrites located in layer 1 (L1), a key site of convergence for higher‐order feedback inputs (Abs et al. 2018; Aru et al. 2020; Suzuki and Larkum 2020; Cohen‐Kashi Malina et al. 2021; Hartung and Letzkus 2021). This reduction in excitability impairs the generation of somatic action potentials in response to feedback signals, effectively decoupling distal dendritic excitation from somatic integration. As a result, functional communication between L5 PN compartments is disrupted, thereby interrupting reverberatory activity within thalamocortical loops.

Pharmacological inhibition of the posteromedial thalamic nucleus (POm) with muscimol causes decoupling of layer 5 pyramidal neurons (L5 PNs), supporting the idea that higher‐order thalamic input enhances dendritic‐somatic coupling via mGluR signaling. Indeed, the dendrosomatic decoupling of L5 PNs is resistant to antagonism of NMDA, AMPA, and GABA_A_ receptors, yet can be induced by local application of muscarinic antagonists (e.g., atropine) or metabotropic glutamate receptor (mGluR) antagonists (e.g., MCPG) to the apical dendrites during wakefulness (Suzuki and Larkum 2020).

While anesthetics such as isoflurane appear to disrupt higher‐order sensory feedback to the distal dendrites of layer 5 pyramidal neurons (L5 PNs), contributing to cortical disconnection, the ion channels mediating L5 inhibition remain unidentified. As previously noted, neither NMDA nor GABA_A_ receptors appear to underlie the reduction in excitability induced by general anesthetics. Instead, dendritic integration between L5 PN subcompartments has been proposed to depend on potassium channels (Harnett et al. 2013), with the voltage‐gated potassium channel Kv1.2 (Sheng et al. 1994) emerging as a particularly compelling candidate due to its high expression in L5 neurons. Supporting this hypothesis, volatile anesthetics have been shown to potentiate Kv1.2 channels (Liang et al. 2015) through a molecular mechanism involving multiple, concentration‐dependent binding sites (Stock et al. 2017), potentially accounting for their complex modulatory effects on neuronal excitability.

To further elucidate the role of voltage‐gated potassium channels in the inhibition of neocortical neurons by general anesthetics, we investigated the effects of sevoflurane on layer 5 pyramidal neurons. Sevoflurane is a volatile anesthetic with slightly lower potency than isoflurane (Sonner et al. 2003), characterized by rapid induction and emergence as well as a non‐irritating odor—features that have contributed to its widespread clinical use (Sakai et al. 2005; Sahu et al. 2011). Previous studies have demonstrated that sevoflurane potentiates voltage‐gated Kv1.2 channels in a dose‐dependent manner (Barber et al. 2014; Covarrubias et al. 2015; Liang et al. 2015; Stock et al. 2018), making it a useful pharmacological tool to assess the contribution of Kv1.2 to anesthetic‐induced neuronal inhibition. We examined the low‐dose effects of sevoflurane (∼22 μM) on the membrane properties of L5 PNs in the primary auditory cortex of adult mice using whole‐cell patch‐clamp recordings. The auditory cortex is particularly suitable for this investigation as it remains responsive to auditory stimuli under anesthesia (Banks et al. 2018; Bergman et al. 2022), yet does not support conscious auditory perception (Krom et al. 2020), thereby enabling the study of graded shifts in neuronal activity rather than complete silencing.

Anticipating our findings, sevoflurane significantly reduced the firing frequency of L5 PNs and elicited cell‐type‐specific changes in intrinsic membrane properties, here designated type A and B according to electrophysiological profiles (Lee et al. 2014; Hilscher et al. 2017). In type A neurons, it depolarized the resting membrane potential, whereas in type B neurons, it increased input resistance and altered action potential (AP) parameters, including reduced amplitude and increased half‐width. The application of TsTX‐Kα, a selective Kv1.2 channel blocker, partially reversed these effects at subthreshold potentials (−50 mV), implicating Kv1.2 potentiation as a contributing mechanism in the suppression of L5 PN excitability. Additionally, NEURON simulations using a biophysically detailed model of thick‐tufted L5b pyramidal neurons (type A PNs) showed a statistically significant increase in persistent K^+^ conductance under sevoflurane, further supporting the involvement of Kv1.2 activation in modulating excitability. Together, these findings provide novel insights into the molecular actions of sevoflurane and underscore the role of Kv1.2 channels in the anesthetic modulation of cortical pyramidal neuron activity.

## 2 Methods

### 2.1 Animals

A total of 40 adult C57BL/6 (Fundação Edson Queiroz, Universidade de Fortaleza, Brazil) wild‐type mice of either sex, aged 4–9 weeks old (*n* = 37 pyramidal neurons with stable recordings), were used for this study following the guidelines of the local university Ethics Committee in the Use of Animals of the Federal University of Rio Grande do Norte and approved by ethics protocol 064/2018, CERTIFICADO n° 135.064/2018. Mice of weight > 25 g were housed in ventilated microisolator cages (dimensions 37.9 × 19.7 × 12.7 cm) with 3–6 companions, with food and water *ad libitum* on a 12 h/12 h light/dark cycle.

### 2.2 Electrophysiology

Mice were sacrificed by intraperitoneal injection of ketamine (500 mg/kg) and perfused by ice‐cold standard artificial cerebrospinal fluid containing (aCSF, in mM): NaCl, 119; KCl, 2.5; NaH2PO4, 1.25; NaHCO3, 24; HEPES, 5; Glucose, 12.5; MgSO4.7H2O, 2; CaCl2.2H2O, 2 (from Sigma Aldrich, MO, USA). Next, brains of mice were rapidly dissected, glued to a platform and submerged in ice‐cold sucrose aCSF containing (in mM): KCl, 2.49; NaH2PO4, 1.43; NaHCO3, 26; Glucose, 10; Sucrose, 252; MgSO4.7H2O, 4; CaCl2.2H2O, 1. Next, 4 slices of 300 μm thickness were routinely collected and hemisected to obtain 8 slices containing the region of the primary auditory cortex. Slices were next transferred to a submerged chamber and incubated in standard artificial cerebrospinal fluid at 33°C for 30 min; next, slices were kept at room temperature (23°C–25°C). Patch pipettes from borosilicate glass capillaries (GC150F‐10, Harvard Apparatus, MA, USA) were pulled on a vertical puller (PC‐10, Narishige, Japan). Pipettes were filled with internal solution containing (in mM): K‐gluconate, 130; NaCl, 7; MgCl_2_, 2; ATP, 2; GTP, 0.5; HEPES, 10; EGTA, 0.1 (from Sigma Aldrich, MO, USA). The pH was adjusted to 7.2 using KOH. Whole‐cell current‐clamp and voltage clamp recordings were made in room temperature (23°C–25°C) and acquired using an Axopatch 200B amplifier (Axon Instruments, CA, USA), and signals were digitized with a BNC‐2111 panel block (National Instruments, TX, USA). The primary auditory cortex was recognized based on the shape of the hippocampus and the rhinal fissure of the lateral surface. The fifth layer was estimated by the distance from the internal cortical edge and the density and size of PN soma. Healthy pyramidal cells were identified by their pyramidal‐like shape and cytoskeletal integrity identified as three‐dimensional features such as lighter and darker edges under Differential Interference Contrast (DIC) microscopy, and routinely clamped to −67 mV, a holding potential closer to the resting of L5 PNs (Lee et al. 2014). WinWCP software implemented by Dr. J. Dempster (University of Strathclyde, Glasgow, UK) was used to record electrophysiological responses in current and voltage clamp. Pipette resistances varied from 6 to 8 MΩ (access resistance was routinely compensated by 70%). Cells with a resting membrane potential more depolarized than −45 mV after breaking in were excluded from further analysis.

### 2.3 Pharmacology

Sevoflurane (Sevocris, 100%, 1 mL/mL, Cristália Produtos Químicos Farmacêuticos Ltda, Itapira‐SP, Brazil) was delivered to the recording chamber after solubilizing in aCSF by passing the carbogen mixture (95% O_2_, 5% CO_2_) gas line through a vaporizer (Midmark, OH, USA, pre‐calibrated for isoflurane), in initial experiments at 1%, and next routinely at 0.5% as this flow was sufficient for electrophysiological effects on pyramidal cells. The peristaltic pump speed was set to 2–5 mL/min. The aCSF containing sevoflurane was applied through a line of polytetrafluoroethylene (PTFE) tubing, as sevoflurane can adhere to standard silicone tubing. Thereby, two bath lines were used for each experiment: one for standard oxygenated aCSF and a second line for oxygenated aCSF with additional sevoflurane (bubbled into aCSF solution for 20–40 min before delivery to the recording chamber). The washout recordings were performed 10–20 min after switching back to the aCSF bath line. In some experiments, the Kv1.2 channel blocker rTityustoxin‐Kα (TsTX‐Kα, Alomone Labs, Cat#: STT‐360, 100 nM) was added to aCSF pre‐bubbled with sevoflurane.

### 2.4 Gas Chromatography–Mass Spectrometry

Sevoflurane concentration was measured in the aCSF using the gas chromatography–mass spectrometry (GC–MS) technique (Figure S1). Sevoflurane concentrations were measured from the aCSF flask before being pumped through PTFE tubing, as well as from the recording chamber (500 μL collected into gas‐tight flasks of 2 mL). The samples were then mixed with an equal volume of pure hexane and vortexed to facilitate the extraction of sevoflurane from the aqueous phase into the non‐aqueous hexane phase. After separating the phases, a portion of the non‐aqueous phase was transferred to insert tubes and stored at room temperature overnight before quantification. GC–MS analyses were performed using an Agilent 5977C GC/MSD single quadrupole mass spectrometer coupled to an Agilent 8860 gas chromatograph (Agilent, Stockport, UK). The sevoflurane standard was diluted with hexane (analytical grade, Merck) to construct the calibration curve at concentrations of 1, 2, 5, 12.5, 25 and 50 nL/mL. The auto‐sampler was equipped with a 10 μL syringe, which was cleaned with isopropanol for two and ten cycles before and after injection, respectively. The sample was pumped six times before the injection of 1 μL, with a syringe filling rate of 300 μL/min. An HP q ms column (30 m, 0.25 mm internal diameter, 0.25 m film thickness, Agilent, Stockport, UK) was used in the analyses with a helium gas flow of 1.2 mL/min. The column inlet line was maintained at 80°C, and the splitless injection mode was chosen. The oven was kept at 40°C for 5 min and then heated at a rate of 50°C/min up to 200°C, where it was held for 2 min. The transfer line was maintained at 200°C. The mass spectrometer was operated in electron ionization (EI) mode with the quadrupole temperature at 150°C, source temperature at 250°C, and an energy of 70 eV. The mass spectrum was collected between 50 and 500 m/z (molecular mass per ion), with an acquisition period of 0 to 10 min. MassHunter software (Agilent, Stockport, UK) was used for data analysis and equipment control. The response was evaluated based on the peak area identified in the Total Ion Count (TIC) chromatograms. The analyte was identified by comparing the retention time of the standard and the NIST (National Institute of Standards and Technology, Gaithersburg, MD, USA) spectral library. The amount of sevoflurane in the aqueous solution was determined after three liquid–liquid extractions with an equal volume of hexane to remove all dissolved volatile anesthetic from the aqueous fraction. The partition coefficient for sevoflurane (between 2.41 and 3.18) enabled this extraction strategy and 0.5 μL aliquots were injected into the equipment.

### 2.5 Data Analysis

We utilized custom‐written Python scripts to analyze electrophysiological parameters. The resting membrane potential was calculated as the average membrane potential during the first 100 ms before the application of a current step protocol (−100 to 450 pA, 1 s duration). Input resistance was determined using Ohm's law based on the current response to a −5 mV step. The action potential threshold was identified from the first action potential where the change in membrane potential exceeded 20 mV/ms. Rheobase was calculated as the amount of current required to elicit the first action potential following a ramp protocol (−50 to 200, 300, 500 or 700 pA, duration 500 ms). The action potential amplitude was defined as AP threshold to peak and action potential half‐width was measured at the half peak amplitude (peak minus threshold). Steady‐state hyperpolarization was calculated as the difference between the resting potential and the membrane potential during the final 50 ms of a −100 pA step. Sag was defined as the difference between the minimum membrane potential and the steady‐state potential during the last 50 ms of a −100 pA step. Afterdepolarization (ADP) was calculated as the difference between the resting potential and the maximum membrane potential during the 200 ms following a −100 pA current step. Afterhyperpolarization (AHP) was calculated as the difference between the resting potential and the lowest membrane potential during the 200 ms after a 250 pA step. The criteria for subdivision of PNs into type A or type B were based on the electrophysiological profile (Lee et al. 2014), specifically the summed magnitude of the sag, ADP and AHP in response to a −100 and +250 pA step larger than 6.5 mV (Hilscher et al. 2017; Nogueira et al. 2023). Firing frequency was calculated as the total number of action potentials in a 1 s protocol in response to different positive current steps, as initial/max frequency and steady state frequency were not applicable after sevoflurane administration.

### 2.6 Pyramidal Neuron Model

Type A pyramidal neurons (PNs), corresponding to thick‐tufted layer 5b pyramidal neurons, were simulated using a biophysically detailed and morphologically realistic model adapted from Hay et al. (2011). Specifically, we employed the third model variant described by Hay, which integrates experimental constraints from both somatic step‐current responses and backpropagating action potentials (bAPs), thereby providing a physiologically accurate representation of neuronal excitability.

The liquid junction potential (JP) was not corrected in the experimental data; however, for the pyramidal neuron model, the JP correction value was calculated from our ionic composition of internal and external solutions used in the patch‐clamp experiments (−14 mV). Figures S2 and S3 displays both experimental and simulated data with junction potentials correction.

Our electrophysiological experiments were performed under whole‐cell current‐clamp conditions. Because of inherent space‐clamp limitations, stimulation primarily affected ion channels in the soma, proximal dendrites, and axon initial segment. Although the NEURON model allows multicompartmental simulations of dendritic trees, our goal was to directly compare the effects of sevoflurane on the somatic compartment—the region most accurately controlled in our recordings. Therefore, to reproduce the experimental conditions when assessing the effects of sevoflurane and/or TsTX‐Kα on intrinsic membrane dynamics, the model was constrained accordingly: with the exception of leak conductance (g_leak), all somatic parameters were allowed to vary, while dendritic parameters remained fixed at their default values defined in the original model. The tunable parameters included calcium‐activated potassium channels (SK); transient and persistent potassium channels (KT, KP); the delayed rectifier potassium channel (SKv3.1); transient and persistent sodium channels (NaT, NaP); and high‐ and low‐voltage‐activated calcium channels (Ca_HVA, Ca_LVAst). Calcium‐related parameters were also allowed to vary, including intracellular buffering (gamma = 0.05: percent of free calcium) and the calcium decay rate. Consistent with the idea that the dendritic compartment plays a limited role in determining sevoflurane's effects on somatic excitability under our recording conditions, convergence analyses (Figures S4 and S5) showed that relaxation of somatic parameters alone produced a substantial minimization of the fitness function during optimization, leaving little potential for further improvement through additional relaxation of dendritic parameters.

Finally, to more accurately capture the steady‐state effects of sevoflurane on Kv1.2 channel gating (Liang et al. 2015), we implemented a uniform leftward shift of −3 mV in the activation curve of the persistent potassium conductance (KP) across all conditions. While this shift produced minimal effects under baseline conditions, it significantly improved the model's fit to electrophysiological traces obtained under sevoflurane and sevoflurane + TsTX‐Kα treatments, supporting its inclusion as a relevant mechanistic parameter.

### 2.7 Real‐Valued, Multi‐Objective, Adaptive Evolutionary Strategy With Elitism Evolutionary Algorithm

We developed an adaptive evolutionary algorithm in Python to optimize conductance‐based neuron models from NEURON by minimizing the discrepancy between simulated and experimental voltage traces. The method integrates real‐valued genetic operators with domain‐specific fitness evaluation (Deb et al. 2002). Parameters for population initialization were sampled by Latin hypercube sampling across physiologically valid bounds from Hay et al. (2011). The initial population comprised 800 individual neurons, a value determined by benchmarking algorithm performance across smaller population sizes. Fitness evaluation consisted of a multi‐component fitness function with quantified model accuracy. First, trace similarity weighted RMS error with a 10× penalty was applied to spike regions. The spike fidelity applied penalties for missing or false spikes, assessed both as binary presence/absence and spike count deviations. For temporal precision, we normalized errors in spike timing (first/last spikes) and inter‐spike intervals (ISIs), calculated using the Wasserstein distance between simulated and experimental ISI distributions for the genetic operators, in tournament selection with an adaptive size of 4%–10% of the population. Generation crossovers were done by BLX‐α blending (Corne and Lones 2018) with an *α* = 0.5 and expanded bounds to promote diversity including Gaussian perturbation with cooling schedule (*σ* ∝ generation^−1^). The adaptive mechanism uses dynamic rates with mutation and crossover probabilities adjusted per generation with higher probabilities at early stages and lower at later stages. The diversity preservation was added to improve the fitness of the traces. The top 10% of the population were preserved in each generation; when diversity fell below 1% of the best fitness, random neuron individuals were added to the tournament. The termination criteria were (1) if the maximum number of generations reached 35, or (2) if any improvement was less than 0.1% for over 5 generations, which generated an early stop.

### 2.8 Statistical Analysis

Sample size was calculated using R with One‐tail Analysis of Variance (ANOVA), with the parameters: power = 80%; effect size = 0.8; *p* = 0.05; *n* = 6, with a minimum sample size of 30 neurons (5 per group). Assessment of data distribution using the Shapiro–Wilk test confirmed that many electrophysiological properties (e.g., input resistance, afterhyperpolarization, firing frequency) were not normally distributed (*p* < 0.05). Consequently, for comparisons between independent groups (type A vs. type B neurons), the non‐parametric Mann–Whitney *U* test was used. To estimate the effect of sevoflurane and TsTX‐Kα, while accounting for within‐cell dependencies, we fitted a series of linear mixed‐effects models separately for type A and type B neurons. For each parameter, a model was specified with the variable as the dependent outcome and sevoflurane condition as a fixed effect, with random intercepts for each cell to account for repeated measurements.

Model fitting was performed using the Nelder–Mead optimization algorithm with increased iteration limits to ensure convergence. For each coefficient, we extracted parameter estimates, standard errors, Wald *z*‐scores, and two‐sided *p*‐values. To control for multiple comparisons across all variables, false discovery rate (FDR) adjustment was applied using the Benjamini–Hochberg procedure. Significance was assessed based on the adjusted *p*‐values, and 95% confidence intervals were derived from the model estimates. Model fitting and inference were implemented using the statsmodels library in Python (scipy.stats, SciPyversion 1.16.1). To compare current–voltage (I–V) relationships across experimental groups, we performed linear regression on per‐cell voltage‐clamp recordings within defined voltage segments. For each cell, subthreshold current responses were extracted over two voltage intervals: −77 to −62 mV and −62 to −47 mV. For each group and neuron subtype, linear regressions were independently fit to current as a function of the recorded voltage using ordinary least squares. Group‐level trends were visualized by plotting the mean slope across cells, along with a 95% confidence interval derived from the standard error of the mean. To test for statistically significant differences in both current amplitude and slope between experimental conditions, we utilized a series of mixed effects linear models. For current amplitude, data were grouped into 5 mV voltage bins for comparison across all conditions and neuron types. For slope calculations, the data were divided into two distinct voltage segments as previously described. The mixed effects linear model approach was chosen to appropriately account for the nested structure of the data (multiple measurements per cell) and allow for both fixed effects (e.g., drug condition) and random effects (e.g., cell‐to‐cell variability). To facilitate interpretation, the results of the models were visualized using *z*‐scores and P_FDR_ values. Horizontal reference lines at −log_10_ (P_FDR_) thresholds of 1.3 and 2 were added to denote significance at the false discovery rate (FDR) corrected thresholds of *p* = 0.05 and *p* = 0.01, respectively.

For the computer simulation data, unpaired comparisons (aCSF vs. Sevo and aCSF vs. Sevo + TsTX) were analyzed using the Mann–Whitney *U* test since these involve independent samples. Analyses were implemented in Python 3.9 using scikit‐learn for linear regression and scipy.stats for non‐parametric testing. Data visualization was performed using matplotlib. For the NEURON model, a non–parametric Mann–Whitney *U* test was used to assess statistical significance. No outlier test was conducted for experimental or computational analyses.

## 3 Results

**FIGURE 1:**
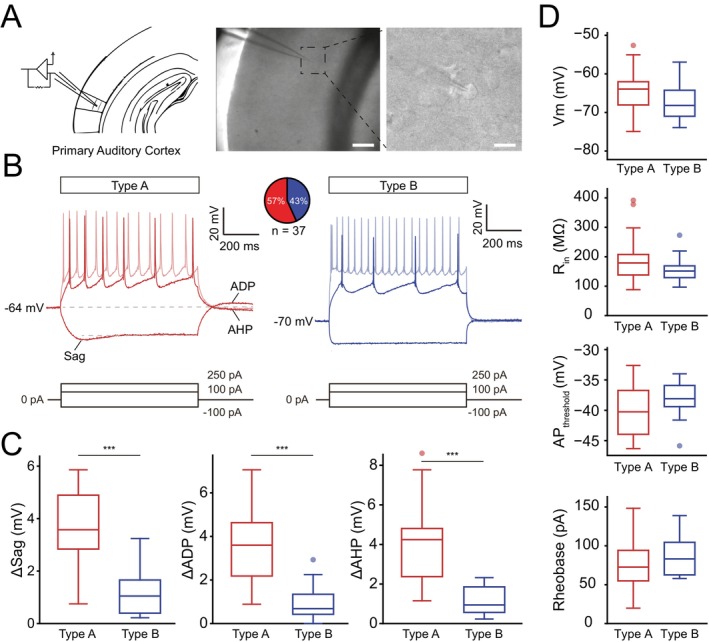
Subclassification of L5 pyramidal neurons according to membrane properties. (A) Schematic drawing of the patch pipette in layer 5 (L5) of the auditory cortex, inset patched neuron. (B) Representative firing properties of a type A (*n* = 21 neurons) and a type B (*n* = 16 neurons) L5 pyramidal neuron (PN) in response to negative and positive current injections. (C) Description of electrophysiological properties used for neuron classification: sag (*p* = 2.1e‐5), afterdepolarization (ADP, *p* = 1.2e‐5), afterhyperpolarization (AHP, *p* = 2.1e‐5) magnitude. (D) Resting membrane potential (V_m_), input resistance (R_in_), action potential (AP) threshold and rheobase current are not useful for subclassification. Boxplots show median (horizontal bar), interquartile range (IQR, box), and 1.5× IQR values. Statistical significance shown by **p < 0.01, ***p < 0.001. Full statistical report in Table S1.

Here, our aim was to investigate the potential role of voltage‐gated potassium channels in the inhibition of neocortical neurons by general anesthetics. To this end, we started by investigating the cellular effects of the volatile anesthetic sevoflurane on layer 5 pyramidal neurons (L5 PNs, *n* = 37) from coronal slices containing the primary auditory cortex (Figure 1A) of adult C57BL/6 mice.

### 3.1 Cellular Effects of the Volatile Anesthetic Sevoflurane on Layer 5 Pyramidal Neurons

The auditory cortex was chosen due to being less affected by anesthetics compared to other functional cortical areas (Banks et al. 2018), thereby allowing the study of gradual membrane effects of sevoflurane. We collected passive and active membrane properties from PNs with stable resting potential and input resistance for pharmacological manipulations. The electrophysiological membrane response to small negative and positive voltage steps was used to define the subdivision of L5PNs into type A and type B PNs (Lee et al. 2014; Hilscher et al. 2017). It has previously been shown that type A L5PNs correspond to deeper L5b, thick‐tufted, cortico‐thalamic or cortico‐collicular PNs, while type B cells correspond to superficial L5a, thin‐tufted, cortico‐callosal projecting PNs (Lee et al. 2014; Joshi et al. 2015; Hilscher et al. 2017). In our sample, we confirmed that type A L5 PNs can be classified by presence of a membrane sag, ADP, and AHP, while the absence of sag, ADP, and AHP indicate type B PNs (Figure 1B,C), but cannot be separated based on resting membrane potential (type A: −64.26 ± 1.24 mV; type B: −67.29 ± 1.35 mV), input resistance (type A: 196.85 ± 18.20 MΩ; type B: 157.92 ± 11.10 MΩ), AP threshold (type A: −39.97 ± 0.99 mV; type B: −37.94 ± 0.81 mV) or current needed to elicit the first AP in a ramp protocol (Rheobase, type A: 75.08 ± 7.66 pA; type B: 86.76 ± 6.53 pA, Figure 1D, Table S1). This categorization of L5 PN main subtypes was next used to determine whether the general anesthetic sevoflurane exerted distinct effects on the two identified L5 PN subtypes.

**FIGURE 2:**
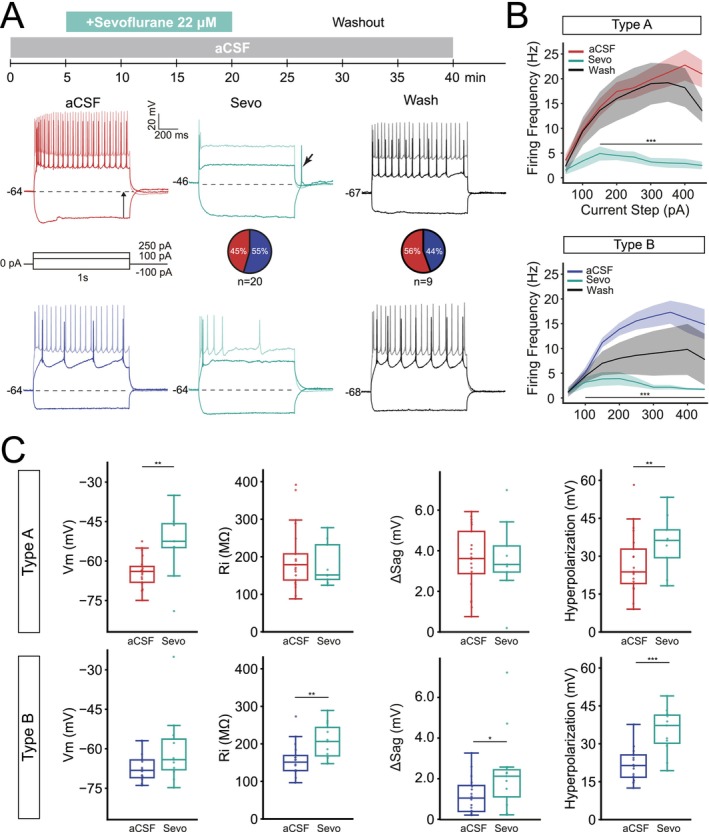
Subtype specific effect of low concentration sevoflurane on L5 PNs. (A) Representative current clamp recordings in response to a hyperpolarizing and two depolarizing current steps before (artificial cerebrospinal fluid—aCSF), during (15 min in bath) and after washing out of sevoflurane (22 μM) for type A (top; sevo, *n* = 9 neurons; washout, *n* = 5 neurons) and type B (bottom; sevo, *n* = 11 neurons; washout, *n* = 4 neurons) PNs. Note, fewer cells remained in good quality after 40 min. Arrows show hyperpolarization magnitude and rebound AP. (B) Sevoflurane consistently decreased firing frequency in both cell types in response to current steps larger than 150 pA. Firing frequency recovered better in type A cells than in type B after washout. (C) Boxplots showing sevoflurane significantly depolarizes resting potential of type A cells (*p* = 0.006, *n* = 9 neurons) while increasing input resistance of type B PNs (*p* = 0.002, *n* = 11 neurons). Application of sevoflurane also increased the Δsag value for type B PNs while hyperpolarization in response to a −100pA increased for type A (*p* = 0.008) and for type B (*p* = 1.4e‐6) L5 PNs. Boxplots show median (horizontal bar), interquartile range (IQR, box), and 1.5× IQR min/max values. Significance levels *p < 0.05, **p < 0.01, ***p < 0.001. Full statistical report in Table S2.

Sevoflurane was administered by continuously bubbling it into artificial cerebrospinal fluid (aCSF) using a vaporizer, in combination with a carbogen gas mixture (95% O_2_, 5% CO_2_), following established protocols (Nishikawa and MacIver 2001; Huskens et al. 2016). The gas mixture was delivered to the recording chamber through gas‐tight polytetrafluoroethylene (PTFE) tubing to ensure consistent anesthetic concentration (Bhattacharji et al. 2010; Huskens et al. 2016). The concentration of sevoflurane dissolved in aCSF was quantified by calibration curves from serial dilutions of sevoflurane in hexane and analysis by gas chromatography–mass spectrometry (Figure S1). We first collected samples in gas‐tight vials from the aCSF flask bubbled with 0.5% of sevoflurane in pressurized carbogen mixture at 8 time points (5–40, 5 min interval) to determine equilibrium concentration in aCSF (Figure S1). Samples were also collected from the recording chamber for different vaporizer settings of sevoflurane into the carbogen flow after equilibrium (Figure S1). Concentrations of collected samples were extrapolated from the calibration curve and a stable concentration of sevoflurane in the aCSF flask was observed after 40 min (22.7 μM, Figure S1), which was similar to the recording chamber concentration (22.6 μM, Figure S1) using the same vaporizer settings. Therefore, all experiments had sevoflurane bubbled into the aCSF for 40 min before starting electrophysiological recordings and we found that 0.5% vaporizer setting generated a sufficient concentration (~22 μM) of sevoflurane to inhibit auditory cortex L5 PNs firing (Figure 2A).

Pyramidal neurons of the auditory cortex were gradually inhibited by sevoflurane (delay 5–15 min) and in general only showed one or a few APs in response to 100 pA current injection after 15 min bath application of sevoflurane (Figure 2A,B). In a few cells, a rebound action potential could be observed (3/21 type A, 1/16 type B PNs). For both cell types firing frequency was significantly reduced by sevoflurane (at steps from +150 to +450 pA, *p* < 0.001). Firing frequency increased again after 10–20 min of washout with aCSF but failed to reach higher firing frequency in response to 350–450 pA steps in type A PNs (Figure 2A,B). Type B PNs recovered less after washout than type A PNs (Figure 2B). Looking at basic membrane properties, we found type A PNs to show a significantly depolarized resting membrane potential in the presence of sevoflurane (A_aCSF_ −64.26 ± 1.24 mV vs. A_Sevo_ −53.19 ± 4.29 mV, *p* = 0.006) while it did not alter the resting potential for type B PNs (Figure 2A,C, Table 2). Sevoflurane unexpectedly increased type B PN input resistance (B_aCSF_ 157.92 ± 11.10 MΩ vs. B_Sevo_ 206.60 ± 14.53 MΩ, *p* = 0.002). Sevoflurane application also increased the membrane sag in type B PNs (B_aCSF_ 1.18 ± 0.23 vs. B_Sevo_ 2.33 ± 0.61 mV, *p* = 0.02, Figure 2C) and hyperpolarizing response to a negative current injection (Figure 2A,C) significantly for both cell types (*p* < 0.01, Figure 2C, Tables 1 and 2). Thus, our data show discrepancies in how sevoflurane decreases firing in type A and type B L5 PNs.

**TABLE 1:** Membrane and firing properties of L5 type A PNs before and after sevoflurane application (aCSF, *n* = 21 neurons; sevo, *n* = 9 neurons; washout, *n* = 5 neurons).

	Type A L5 PNs			Sevo vs. aCSF, *p*
	aCSF *n* = 21	Sevoflurane *n* = 9	Washout *n* = 5	
Vm (mV)	−64.26 ± 1.24	−53.19 ± 4.29	−58.49 ± 3.52	0.006*
Rin (MΩ)	196.85 ± 18.20	179.96 ± 19.04	189.40 ± 29.82	0.10
AP Threshold (mV)	−39.97 ± 0.99	−25.54 ± 4.52	−33.38 ± 2.30	3.1e‐5*
Rheobase (pA)	75.08 ± 7.66	81.42 ± 17.93	90.46 ± 25.67	0.71
AP Amplitude (mV)	78.43 ± 1.77	69.71 ± 3.84	71.02 ± 3.28	0.001*
AP Half‐width (ms)	1.71 ± 0.11	2.09 ± 0.15	2.23 ± 0.29	0.10
Δsag (mV)	3.56 ± 0.33	3.58 ± 0.62	2.27 ± 0.82	0.27
Hyperpolarization (mV)	26.83 ± 2.60	35.04 ± 3.76	26.74 ± 3.43	0.008*
ΔADP (mV)	3.58 ± 0.33	2.82 ± 0.74	1.83 ± 0.56	0.30
ΔAHP (mV)	4.02 ± 0.43	4.16 ± 0.73	2.56 ± 1.02	0.96
FF at 50 pA (Hz)	3.62 ± 1.11	1.78 ± 1	2.40 ± 1.50	0.35
FF at 150 pA (Hz)	14.24 ± 1.77	4.78 ± 1.59	13.60 ± 3.75	0.001*
FF at 250 pA (Hz)	18.19 ± 2.14	3.78 ± 1.15	17.60 ± 4.60	6.1e‐5*
FF at 350 pA (Hz)	21.24 ± 2.85	2.78 ± 1.05	19.20 ± 3.83	3.8e‐5*
FF at 450 pA (Hz)	21.00 ± 2.74	2.45 ± 0.83	13.60 ± 2.38	3.4e‐7*

*Note:* Linear mixed‐effects models (random intercepts) with *p*‐values after Benjamini–Hochberg false discovery rate correction. Mean ± S.E.M. Statistical tests exclude washout data due to low *n*.

* *p* < 0.05.

**TABLE 2:** Membrane and firing properties of L5 type B PNs before and after sevoflurane application (aCSF, *n* = 16 neurons; sevo, *n* = 11 neurons; washout, *n* = 4 neurons).

	Type B L5 PNs			Sevo vs. aCSF, *p*
	aCSF *n* = 16	Sevoflurane *n* = 11	Washout *n* = 4	
Vm (mV)	−67.29 ± 1.35	−60.38 ± 4.12	−71.53 ± 1.18	0.11
Rin (MΩ)	157.92 ± 11.10	206.60 ± 14.53	184.64 ± 10.40	0.002*
AP Threshold (mV)	−37.94 ± 0.81	−31.30 ± 2.77	−41.92 ± 2.93	0.001*
Rheobase (pA)	86.76 ± 6.53	58.93 ± 5	81.68 ± 14.99	0.01*
AP Amplitude (mV)	82.69 ± 1.62	71.64 ± 2.52	75.25 ± 4.08	4.8e‐4*
AP Half‐width (ms)	1.59 ± 0.07	2.03 ± 0.14	1.73 ± 0.14	0.01*
Δsag (mV)	1.18 ± 0.23	2.33 ± 0.61	1.73 ± 0.37	0.02*
Hyperpolarization (mV)	21.8 ± 1.63	35.03 ± 2.73	24.81 ± 2.74	1.4e‐6*
ΔADP (mV)	0.99 ± 0.21	1.85 ± 0.51	1.14 ± 0.36	0.06
ΔAHP (mV)	1.12 ± 0.18	1.42 ± 0.30	1.18 ± 0.53	0.52
FF at 50 pA (Hz)	1.25 ± 0.5	1.27 ± 0.60	1.20 ± 0.97	0.98
FF at 150 pA (Hz)	11.19 ± 0.97	4 ± 1.50	7.00 ± 2.41	2.6e‐7*
FF at 250 pA (Hz)	15.56 ± 1.76	3.27 ± 1.04	8.60 ± 3.88	4.2e‐10*
FF at 350 pA (Hz)	17.31 ± 2.32	2.18 ± 0.48	9.40 ± 4.78	2.3e‐8*
FF at 450 pA (Hz)	14.88 ± 3.04	1.73 ± 0.24	7.80 ± 5.17	1.3e‐4*

*Note:* Linear mixed‐effects models (random intercepts) with *p*‐values after Benjamini–Hochberg false discovery rate correction. Mean ± S.E.M. Statistical tests exclude washout data due to low *n*.

* *p* < 0.05.

**FIGURE 3:**
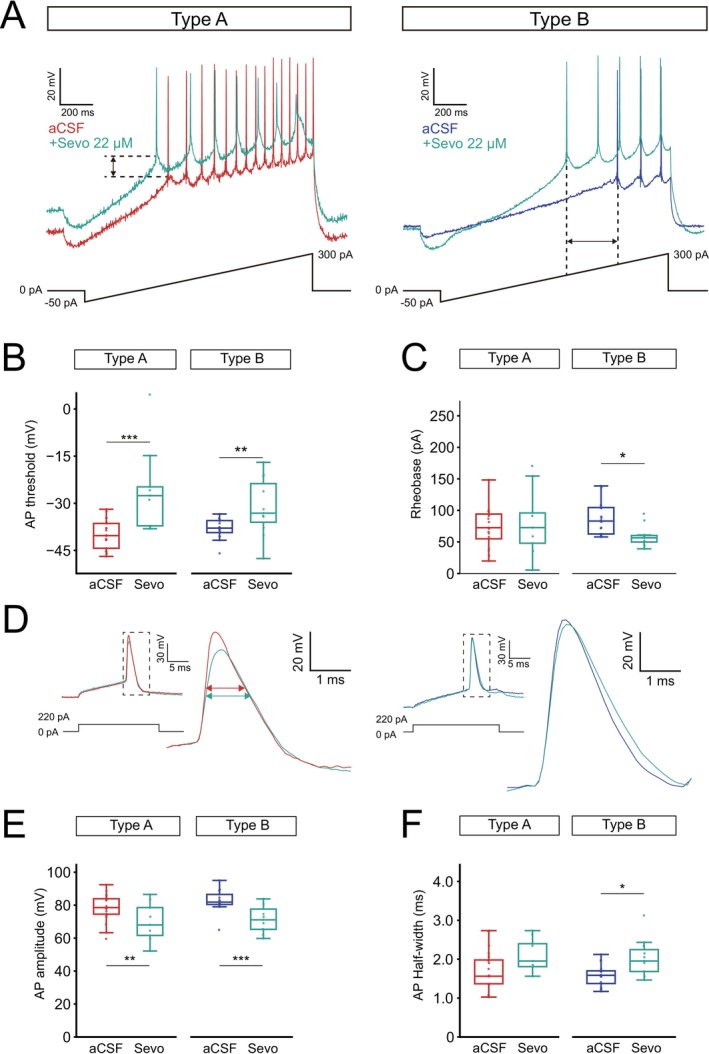
The action potential threshold is depolarized by sevoflurane. (A) Representative voltage traces in response to a ramp protocol before and after sevoflurane application (22 μM) for a type A (sevo, *n* = 9 neurons) and a type B (sevo, *n* = 11 neurons) L5 PN. Dashed lines highlight AP threshold depolarization (left) and decrease in rheobase for type B PNs (right). (B) Boxplots of the mean AP threshold showing significant depolarization in both type A (*p* = 3.1e‐5) and type B PNs (*p* = 0.001). (C) Boxplots of mean current needed to elicit the first AP (rheobase) showed only a significant decrease in type B cell upon sevoflurane application (*p* = 0.01). (D) Representative traces of AP amplitude and half‐width from a type A (red) and a type B (blue) PN before and after sevoflurane application. (E) Boxplots of AP amplitude before and during sevoflurane application for type A and type B PNs. (F) Boxplots showing no alteration of average AP half‐width by sevoflurane for type A PNs while type B PNs showed a widening of the first AP upon sevoflurane application (*p* = 0.01). Boxplot show median (horizontal bar), interquartile range (IQR, box), and 1.5× IQR values. Significance levels *p < 0.05, **p < 0.01, ***p < 0.001. Full statistical report in Table S2.

We continued to examine alterations to AP properties after applying sevoflurane using depolarizing ramp protocols. We found sevoflurane to consistently depolarize the AP threshold in both cell types (A_aCSF_ −39.97 ± 0.99 mV vs. A_Sevo_ −25.54 ± 4.52 mV, *p* = 3.1e‐5; B_aCSF_ −37.94 ± 0.81 mV vs. B_Sevo_ −31.30 ± 2.77 mV, *p* = 0.001, Figure 3A,B), while decreasing rheobase current specifically in type B PNs (B_aCSF_ 86.76 ± 6.53 pA vs. B_Sevo_ 58.93 ± 5 pA, *p* = 0.01, Figure 3C). The AP amplitude in response to a suprathreshold step (220 pA, 20 ms) decreased in both cell types (A_aCSF_ 78.43 ± 1.77 mV vs. A_Sevo_ 69.71 ± 3.84, *p* = 0.001; B_aCSF_ 82.69 ± 1.62 mV vs. B_Sevo_ 71.64 ± 2.52, *p* = 4.8e‐4), while the AP half‐width significantly increased for type B PNs (B_aCSF_ 1.59 ± 0.07 ms vs. B_Sevo_ 2.03 ± 0.14 ms, *p* = 0.01, Figure 3F, Tables 1 and 2). Thereby, despite type B PNs showing higher average input resistance, a more depolarized AP threshold and larger AP half‐width remain aligned with reduced firing in response to sevoflurane.

### 3.2 Implications of Kv1.2 in the Inhibition of Neuronal Excitability

**FIGURE 4:**
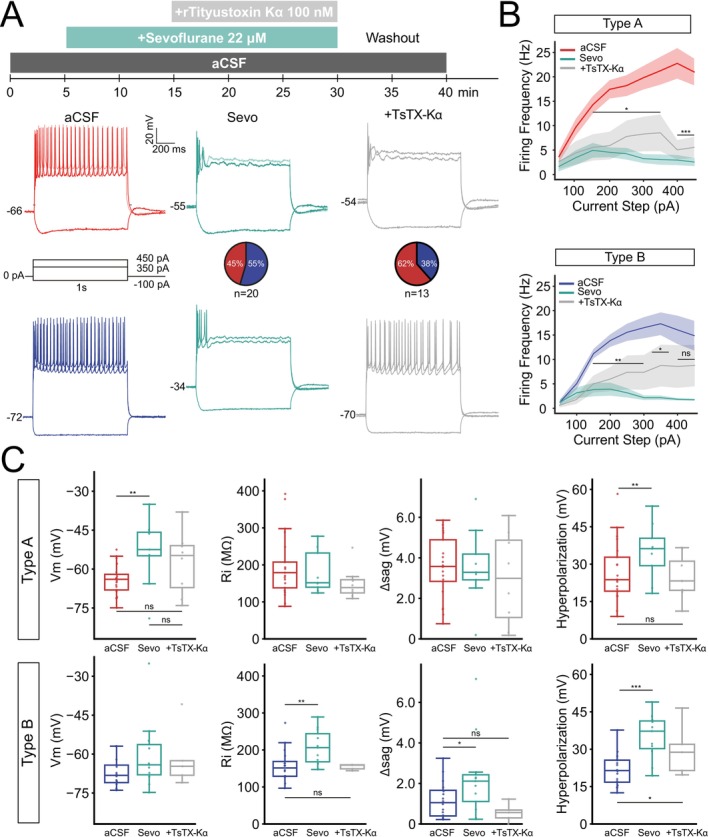
Application of the Kv1.2 antagonist Tityustoxin‐Κα partially decrease effects of sevoflurane. (A) Voltage responses to hyperpolarizing and depolarizing current steps in control conditions, after sevoflurane and after additional Tityustoxin Κα (100 nM, sevo + TsTX‐Κα) application for type A (red; TsTX‐Κα, *n* = 8) and type B (blue; TsTX‐Κα, *n* = 5) L5 PNs. Arrow depicts hyperpolarization magnitude, arrowhead points to the sag or rebound AP. (B) Tityustoxin‐Κα application increased the firing frequency initially decreased by sevoflurane marginally for type A PNs but more so in type B PNs, but could not recover firing frequency to baseline frequency. Asterisks (*) show significant difference between aCSF and sevoflurane + TsTX‐Κα; significance levels *p < 0.05, **p < 0.01, **p < 0.001. (C) Boxplots showing that the average membrane potential depolarization of type A PNs could not be recovered by TsTX‐Κα. (D) Boxplots showing that the average input resistance of type B PNs was recovered by TsTX‐Κα. (E) Boxplots of average Δsag also show TsTX‐Κα to reverse sevoflurane effects. Boxplots show median (horizontal bar), interquartile range (IQR, box), and 1.5× IQR values. Full statistical report in Table S2.

Next, we tested if the physiological effect of sevoflurane was mediated by the modulation of Kv1.2 channels. Here we did not wash out sevoflurane; instead, we added the Kv1.2 channel antagonist Tityustoxin‐Κα (TsTX‐Κα, 100 nM) to the aCSF bubbled with sevoflurane (Figure 4A). We found that sevoflurane‐induced inhibition of firing was sometimes, but not always, counteracted by bath application of TsTX‐Κα to type A PNs (Figure 4A, example of neuron that did not recover firing). In some type B PNs where sevoflurane dramatically depolarized resting membrane potential, application of TsTX‐Κα restored the membrane potential to initial values (Figure 4A, bottom). Quantification of firing frequency in response to increasing current injections showed that TsTX‐Κα partially restored firing frequency; however, the increase was not significantly different from in the presence of sevoflurane alone (Figure 4B, Table 3). The firing frequency between aCSF and sevo + TsTX‐Κα was significantly different for type A PNs (steps 150–450 pA, *p* < 0.05, Table S2) and for type B PNs (150–350 pA, *p* < 0.05, Figure 4B, asterisks, Table S2). Comparison of membrane properties showed that TsTX‐Κα application restored the resting membrane potential, from depolarized values in the presence of sevoflurane, to values similar to (*p* = 0.33, Table S2) aCSF (A_aCSF_ −64.26 ± 1.24 mV; A_Sevo_ −53.19 ± 4.29 mV; A_Sevo+TsTX_ −57.54 ± 4.29 mV; Figure 4C). Type B PN resting membrane potential was not sensitive to sevoflurane nor sevo + TsTX‐Κα. Instead, in type B PNs, the sevoflurane‐induced increase in input resistance was abolished by TsTX‐Κα (Table 3). Moreover, type A PN membrane sag was not altered by sevoflurane and TsTX‐Κα, while for type B PNs the sag response, which had been increased by sevoflurane, could be restored to aCSF values by application of TsTX‐Κα (Figure 4C, Table 3). Lastly, we noticed that the increased hyperpolarization in response to sevoflurane in both cell types was restored to aCSF values by TsTX‐Κα (Figure 4C, Table 3).

**TABLE 3:** Tityustoxin‐Κα (TstX‐Κα, 100 nM) sensitive parameters of L5 type A and type B PNs (type A sevo, *n* = 9 neurons; type A sevo + TsTX, *n* = 8 neurons; type B sevo, *n* = 11 neurons; type B sevo + TsTX, *n* = 5 neurons).

	Type A		Type A PNs sevo vs. sevo + TsTX, *p*	Type B		Type B PNs sevo vs. sevo + TsTX, *p*
	Sevo *n* = 9 mean ± SEM	Sevo + TsTX *n* = 8 mean ± SEM		Sevo *n* = 11 mean ± SEM	Sevo + TsTX *n* = 5 mean ± SEM	
Vm (mV)	−53.19 ± 4.29	−57.54 ± 4.29	0.59	−60.38 ± 4.12	−61.43 ± 5.36	0.87
Rin (MΩ)	179.96 ± 19.04	150.17 ± 15.48	0.99	206.60 ± 14.53	154.29 ± 3.35	0.03*
AP Thres. (mV)	−25.54 ± 4.52	−29.73 ± 4.08	0.59	−31.30 ± 2.77	−38.10 ± 1.34	0.12
Rheobase (pA)	81.42 ± 17.93	119.83 ± 26.00	0.59	58.93 ± 5	90.37 ± 18.84	0.02*
AP Amp. (mV)	69.71 ± 3.84	74.12 ± 3.45	0.54	71.64 ± 2.52	78 ± 5.65	0.42
AP HW (ms)	2.09 ± 0.15	2.11 ± 0.31	0.59	2.03 ± 0.14	1.95 ± 0.32	0.99
Δsag (mV)	3.58 ± 0.62	2.98 ± 0.81	0.59	2.33 ± 0.61	0.56 ± 0.21	0.04*
Hyperpol. (mV)	35.04 ± 3.76	24.83 ± 3.12	0.59	35.03 ± 2.73	29.68 ± 4.78	0.27
ΔADP (mV)	2.82 ± 0.74	2.48 ± 0.71	0.76	1.85 ± 0.51	0.92 ± 0.47	0.22
ΔAHP (mV)	4.16 ± 0.73	3.03 ± 1.04	0.59	1.42 ± 0.30	3.25 ± 0.92	0.02*
FF at 50 pA (Hz)	1.78 ± 1	2.63 ± 1.52	0.59	1.27 ± 0.60	0.40 ± 0.40	0.44
FF at 150 pA (Hz)	4.78 ± 1.59	5.50 ± 2.69	0.72	4 ± 1.50	4.80 ± 1.74	0.71
FF at 250 pA (Hz)	3.78 ± 1.15	7.75 ± 3.26	0.59	3.27 ± 1.04	7.20 ± 3.48	0.24
FF at 350 pA (Hz)	2.78 ± 1.05	8.50 ± 3.76	0.59	2.18 ± 0.48	8.60 ± 4.30	0.13
FF at 450 pA (Hz)	2.45 ± 0.83	5.50 ± 2.15	0.91	1.73 ± 0.24	8.60 ± 4.26	0.22

*Note:* Linear mixed‐effects models (random intercepts) with *p*‐values after Benjamini–Hochberg false discovery rate correction. Mean ± S.E.M. Statistical tests exclude washout data due to low *n*.

* *p* < 0.05.

**FIGURE 5:**
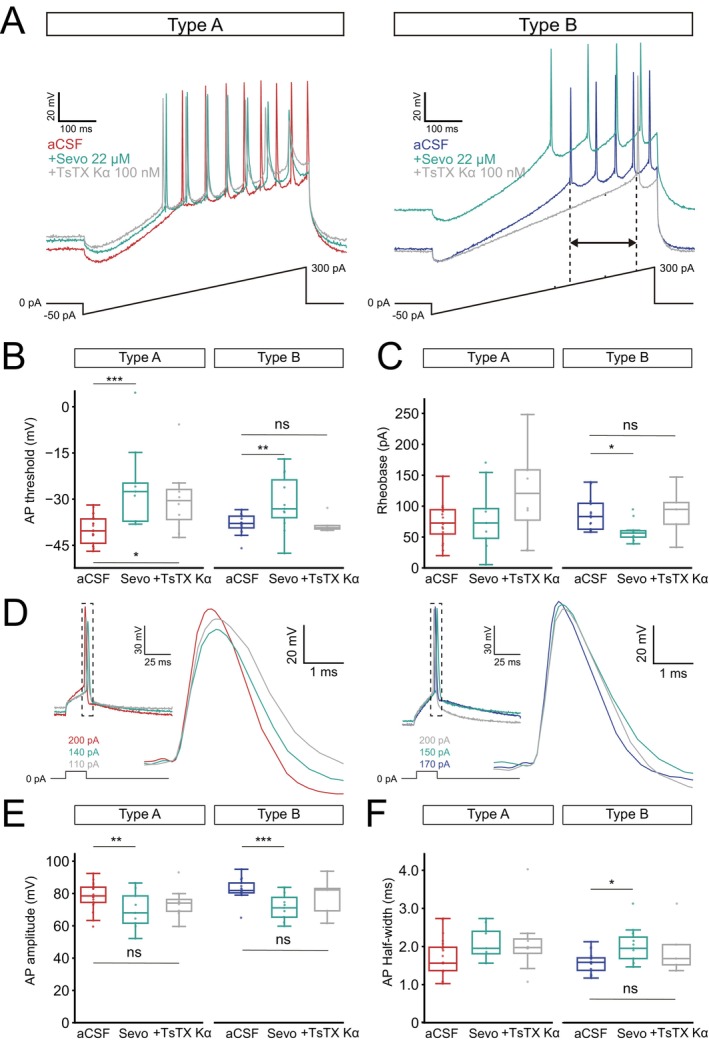
TsTX‐Κα application can restore firing characteristics in type B PNs but less so in type A PNs. (A) Representative voltage traces in response to a ramp protocol before and after sevoflurane (22 μM) and after additional Tityustoxin Κα (100 nM, sevo + TsTX‐Κα) for type A (TsTX‐Κα, *n* = 8 neurons) and type B (TsTX‐Κα, *n* = 5 neurons) PNs. Right: TsTX‐Κα (100 nM) brings back the resting membrane potential. Right: Dashed lines highlight the increase in rheobase. (B) Boxplots of the mean AP threshold showing TsTX‐Κα application can partially restore the lower AP threshold, especially for type B cells. Data for aCSF and after Sevoflurane application are the same as in Figure 3. (C) TsTX‐Κα application restores rheobase to baseline levels for type B PNs, but not for type A PNs. (D) Representative traces of action potential amplitude and half‐width from a type A and a type B PNs in response to different current injections to elicit the first AP in the presence of aCSF (red), sevoflurane (green) and TsTX‐Κα (yellow) for a type A PNs, and a type B RNs (blue‐ aCSF). Higher magnification shows a shift in resting potential. (E) Boxplots of AP amplitude before and during sevoflurane application for type A and type B PNs. (F) Box plots of AP half‐width shows TsTX‐Κα to restore AP half‐width in type B PNs but not type A PNs. Boxplots show median (horizontal bar), interquartile range (IQR, box), and 1.5× IQR values. Significance levels *p < 0.05, **p < 0.01, **p < 0.001. Full statistical report in Table S2.

Examining AP properties in response to ramp protocols showed TsTX‐Κα to hyperpolarize the AP threshold only marginally for type A PNs, although still significantly different from aCSF (*p* = 0.01, Table S2) while returning AP threshold to initial values for type B PNs (B_aCSF_ −37.94 ± 0.8 mV vs. B_Sevo+TsTX_ −38.10 ± 1.34 mV, Figure 5B, Table 3). The decreased rheobase current by sevoflurane application in type B PNs was also restored by TsTX‐Κα application (B_aCSF_ −86.76 ± 6.53 pA vs. B_Sevo+TsTX_ 90.37 ± 18.84 pA, Figure 5C, Table 3). TsTX‐Κα application restored AP amplitude of type A PNs and attenuated the sevoflurane‐induced increase in AP half‐width for type B PNs, indicating that modulation of the action potential shape by sevoflurane is partially mediated through Kv1.2 channel regulation.

**FIGURE 6:**
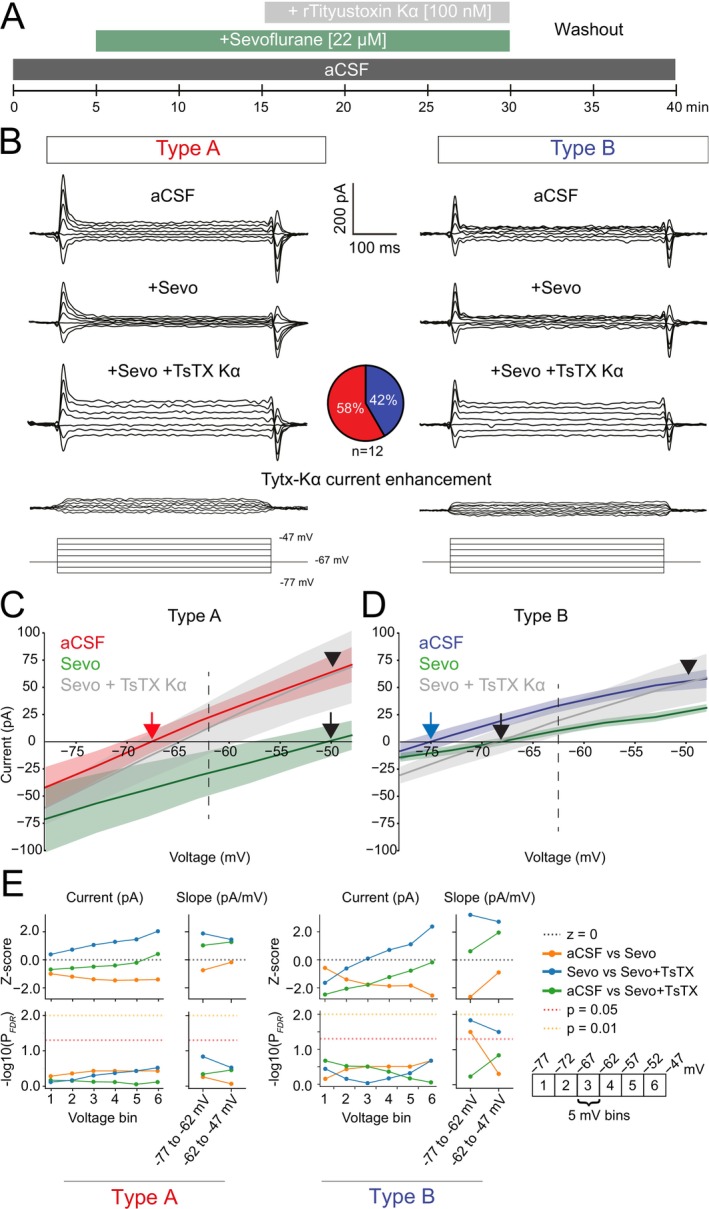
Sevoflurane shifts Kv1.2 activation to more hyperpolarized voltages, suppressing subthreshold currents in L5 pyramidal neurons. (A) Pharmacology schematics. (B) Current responses to 7 subthreshold voltage steps (−77 to −47 mV, 5 mV increments, 500 ms duration) of type A (all groups, *n* = 7 neurons) and type B (all groups, *n* = 5 neurons) PNs in the presence of aCSF, sevoflurane and sevoflurane + Tityustoxin‐Kα. Bottom: The digitally subtracted trace of sevoflurane trace from the Sevo + TsTX‐Κα trace, showing the TsTX‐Κα current enhancement was similar for the two subtypes. (C) Current voltage‐dependency (I–V) plots for average steady state currents (*I*_*ss*_) in response to subthreshold voltage steps in aCSF, after application of sevoflurane and after additional application of Tityustoxin‐Kα (TsTX‐Kα). Arrows denote reversal potential of subthreshold currents in the presence of aCSF (type A—red, type B—blue) and sevo (black). Arrowheads show no difference in average current in response to a −50 mV step in aCSF vs. Sevo + TsTX‐Κα. (D) I–V plots as in ‘C’ for type B PNs. (E) *Z*‐scores and −log_10_ (P_FDR_) plots from mixed effects linear model for each 5 mV voltage bin (1: −77 to −72 mV; 6: −52 to −47 mV) for current amplitudes, and 15 mV bin comparisons (−77 to −62 mV and −62 to −47 mV) for I–V slope analysis. Dotted lines indicate standard significance thresholds and the line at *z* = 0, which represents no effect of the treatment. All error bars represent SEM. Full statistical report in Table S2.

To examine the effect of sevoflurane on subthreshold membrane currents we applied voltage steps (−77 to −47 mV, 5 mV increments, 500 ms) in the presence of aCSF, sevoflurane and sevo + TsTX‐Kα (100 nM) to type A and B PNs (Figure 6A,B). Plotting the average current voltage‐dependency curve showed that sevoflurane shifted the reversal potential of subthreshold membrane currents from −66.59 to −50.33 mV for type A PNs, and from −75.06 to −67.64 mV for type B PNs (Figure 6C,D, red/blue vs. black arrows, denoting type A/B and sevo, respectively). In both subtypes, sevoflurane showed a trend in decreasing steady‐state current amplitudes, with a stronger suppression from −67 to −47 mV (Figure 6C–E). TsTX‐Kα application restored average current magnitude at −50 mV for both type A and B PNs (Figure 6C,D, arrow head); however, less so at more hyperpolarized potentials (Figure 6C,D). Thereby, the putative potentiation of Kv1.2 channels by sevoflurane (increased K+ outflux) may counteract Na + influx at subthreshold potentials in L5PNs, generating decreased current magnitude recorded in the presence of sevoflurane (Figure 6B–D). Interestingly, for type B PNs, sevoflurane decreased average slope at voltage steps −77 to −62 mV, which was restored by the application of TsTX‐Kα (*p* < 0.05 for both aCSF vs. sevo and sevo vs. sevo + TsTX‐Kα, Figure 6D,E). Together, our results indicate that sevoflurane impedes positive inward current (Na+) close to the AP threshold (~−50 mV) for both type A and type B PNs and that this effect is due to potentiation of Kv1.2 channels.

### 3.3 Simulations Using a Detailed Biophysical Model of Thick‐Tufted L5b Pyramidal Neurons

**FIGURE 7:**
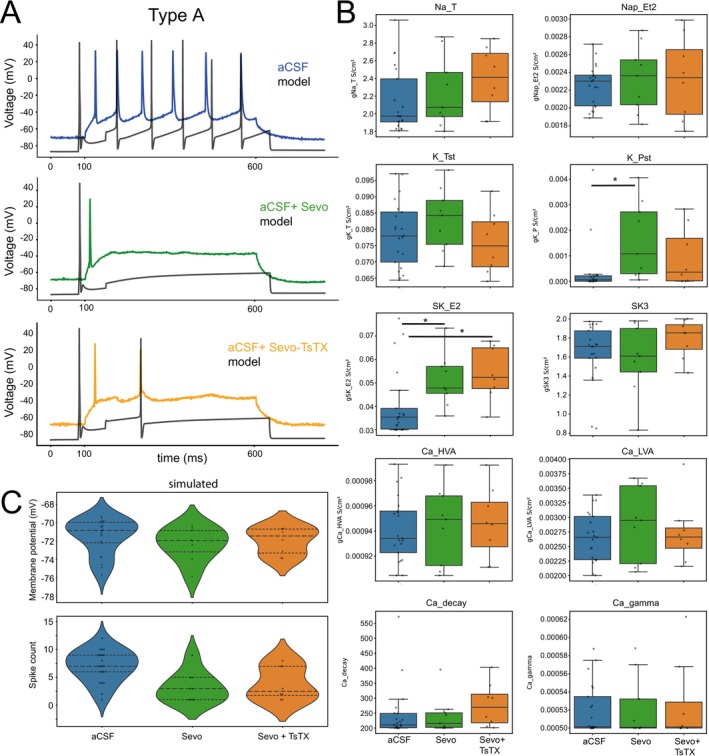
Comparison of experimental data with a prediction model for type A PNs. (A) *Top*: Overlay of experimentally recorded baseline neuronal trace (blue) and model‐predicted trace (black) using estimated channel conductances. *Middle*: Experimental Sevoflurane traces (green) vs. modeled (black) traces after sevoflurane application. *Bottom*: Experimental (yellow) vs. modeled (black) traces under sevoflurane + Tityustoxin‐Kα (Sevo + TsTX‐Kα) conditions. (B) Normalized conductance changes (median ± IQR) for Fast Na's (Na_T), Persistent Na's (Nap_Et2), Fast Kv's (K_Tst), Persistent Kv's (K_Pst), delayed rectifier K^+^ (SKv3_1), calcium‐activated potassium channels (SK_E2); high‐voltage activated Ca^2+^ channels (Ca_HVA), low‐voltage activated Ca^2+^ channels (Ca_LVA), and calcium delay rate and intracellular calcium buffering (gamma). aCSF *n* = 21 neurons, Sevo *n* = 9 neurons, Sevo + TsTX *n* = 8 neurons. Boxplots show median (Q2) (horizontal bar), the box show IQR, Whiskers 1.5 times IQR, full statistical report on Table S3 *p < 0.05. (C) Violin plots show distribution for simulated membrane potential and firing (spike count).

To further investigate the role of sevoflurane in reducing subthreshold currents, we implemented NEURON modeling of the somatic compartment of type A pyramidal neurons, based on a previously published model of large, thick‐tufted layer 5b pyramidal neurons (Hay et al. 2011) and using our experimentally derived parameters recorded at room temperature (25°C). Specifically, we simulated firing frequency under three conditions: aCSF, sevoflurane, and sevoflurane + TsTX‐Kα, using experimental voltage traces as reference data (Figure 7A). The Neuronal Model statistical reports are displayed in Table S3.

Model optimization employed an adaptive evolutionary algorithm designed to minimize discrepancies between simulated and experimental traces by adjusting somatic parameters (Methods). The multi‐component fitness function used in the evolutionary algorithm provides a robust quantification of the model's ability to reproduce key electrophysiological properties of the experimental data across all conditions. As shown in Figures S4 and S5, the fitness function consistently converges during simulations, demonstrating substantial minimization across generations. Since model accuracy is maximized when the fitness value approaches zero, the observed minimization indicates a satisfactory match between experimental and simulated traces given the set of directives and fitness function used in the optimization (Figures S2 and S3). Supporting this conclusion, Table S4 presents a comparative analysis of electrophysiological parameters obtained from patch‐clamp recordings and NEURON simulations of type A pyramidal neuron membrane properties, including resting membrane potential (RMP), spike count, action potential amplitude, and threshold. These electrophysiological metrics were derived from the experimental and simulated traces shown in Figures S2 and S3.

We found that in comparison with aCSF, the sevoflurane application statistically increased overall persistent potassium currents (K_Pst, *p* = 0.0017, non–parametric Mann–Whitney *U* test, Figure 7B and Table S3). The simulations further indicate that co‐application of TsTX‐Kα with sevoflurane reduces the deviation from aCSF observed with sevoflurane alone, suggesting that K_Pst potentiation is attenuated under the combined treatment. This mitigating effect is evident in the data, even though it did not reach statistical significance in the non‐parametric Mann–Whitney *U* test (K_Pst, *p* = 0.0746; Figure 7B and Table S3). The attenuation of sevoflurane effects at subthreshold potentials (−50 mV) by TsTX‐Kα, a selective Kv1.2 channel blocker, implicates Kv1.2 potentiation as a contributing mechanism to the suppression of neuronal excitability. These results reinforce the conclusion that K_Pst modulation—particularly via Kv1.2 channels—plays a robust role in sevoflurane's mechanism of action.

As also shown in Figure 7B, our simulations indicate that sevoflurane's effects cannot be fully explained by K_Pst modulation alone. The model reveals a statistically significant increase in small‐conductance calcium‐activated potassium currents in response to sevoflurane (SK_E2, *p* = 0.009858, non–parametric Mann–Whitney *U* test, Figure 7B and Table S3). Given the good agreement between model and experimental data, the contribution of SK currents appears mechanistically relevant. This finding aligns with a previous report demonstrating that sevoflurane can increase intracellular calcium concentration (Zhu et al. 2021) thereby possibly enhancing SK channel activity, contributing to the central depressant effects of general anesthetics. Relative to aCSF, SK channel conductance was comparable between the sevoflurane and sevoflurane + TsTX‐Kα conditions. This indicates that SK current potentiation by sevoflurane is not mitigated by TsTX‐Kα, consistent with the fact that SK channels are not primary targets of this toxin.

By not showing significant differences in the other modeled conductances (Hay et al. 2011), the simulation results are consistent with our voltage‐clamp data, indicating that subthreshold currents are predominantly increased in the outward direction, impeding the neuron from reaching the AP threshold. This supports the interpretation that Kv1.2 channels contribute to the electrophysiological effects of sevoflurane in type A cells. Additionally, enhanced SK channel activity, potentially arising from indirect (calcium dependent) effects of sevoflurane, is also likely to play a role in its anesthetic action.

The model by Hay et al. is specifically designed to represent large, thick‐tufted layer 5 (L5) type A pyramidal neurons. As noted by Hay et al., extending this model to other cell types would require additional refinements that fall beyond the scope of the present study. Nonetheless, acknowledging that the model's applicability to other neuronal types should be interpreted with caution, we have also carried out simulations for type B cells under aCSF, sevoflurane, and sevoflurane + TsTX conditions, following the same simulation protocol used for type A cells (Figure S6; Table S5). Overall, these simulations reproduce the depressant effect of sevoflurane on type B cells, primarily through significant modulation of persistent potassium currents (K_Pst). Similar to type A cells, our results indicate that alterations in K_Pst alone cannot fully explain the observed effects of sevoflurane, suggesting the involvement of additional ion channels. In type B cells, sevoflurane also induces a marked increase in persistent voltage‐gated sodium currents. However, unlike type A cells, the combined application of sevoflurane and TsTX does not significantly reduce K_Pst in type B cells. Instead, this combination decreases calcium‐dependent parameters of the model, including high‐voltage calcium currents and the calcium decay rate. The mechanisms underlying these differences remain to be elucidated and will require further modeling efforts to be properly addressed.

Although our optimization pipeline successfully reproduced a subset of somatic electrophysiological features, several simulated traces for type A and type B neurons exhibited a sharp depolarizing bump following the first spike (Figures S2 and S3), likely arising from compensatory conductances in adjacent compartments. This artifact, together with residual mismatches in resting membrane potential, spike amplitude, and action potential shape, highlights that numerical convergence of the fitness function does not necessarily guarantee full physiological realism. These discrepancies reflect intrinsic constraints of multi‐compartment optimization, where high model dimensionality can drive the optimizer toward solutions that satisfy the formal objectives while deviating from experimentally observed dynamics. Moreover, because our recordings were obtained at the soma, a simpler point‐neuron model might have provided more interpretable results with fewer degrees of freedom, although at the expense of the morphological detail needed for future extensions. Despite these limitations, the present models consistently captured the relative effects of sevoflurane across experimental conditions—particularly the robust reduction in K_Pst‐mediated conductances—which was the primary objective of our simulations. Taken together, our results support the qualitative conclusions of the study, while also underscoring the importance of refining optimization strategies and exploring simplified neuronal representations in future work.

## 4 Discussion

General volatile anesthetics modulate neuronal activity through diverse and distinct mechanisms (Nishikawa and MacIver 2001; Ogawa et al. 2011; Zhou et al. 2015; Banks et al. 2018). These effects often exhibit cell‐type specificity, with cortical pyramidal neurons generally displaying greater sensitivity to anesthetics than cortical interneurons (Kaneko et al. 2016; Bharioke et al. 2022; Speigel and Hemmings Jr 2022). This variability likely stems from the differential modulation of ion channels by various anesthetic agents (Covarrubias et al. 2015; Kelz and Mashour 2019; Hao et al. 2020). For example, propofol selectively enhances GABAergic transmission (Bastos et al. 2021), while urethane exerts broader effects by enhancing GABA_A_, glycine, and nicotinic acetylcholine (nACh) receptor‐mediated currents and inhibiting NMDA and AMPA receptor‐mediated currents in heterologous expression systems (Hara and Harris 2002). Furthermore, anesthetics such as chloroform and isoflurane activate two‐pore domain potassium (K2P) channels, including TREK‐1, highlighting the ion channel specificity underlying their effects (Pavel et al. 2020).

Despite the expected link between anesthetic modulation of ion channels and cell‐type‐specific effects on neuronal excitability, the specific channels responsible for layer 5 (L5) pyramidal neuron (PN) inhibition remain unclear. Given the critical role of L5 PNs in mediating anesthetic‐induced unconsciousness (Suzuki and Larkum 2020; Bharioke et al. 2022), our study aimed to investigate interactions between sevoflurane and ion channels that might contribute to cell‐specific modulation of these neurons. Specifically, we examined the effects of low‐dose sevoflurane on L5 PNs in the auditory cortex of adult mice, focusing on the contribution of subthreshold potassium currents mediated by voltage‐gated potassium channels to the observed changes in cellular excitability.

Our study presents limitations primarily related to technical aspects of the experimental design. One major constraint was the duration of recordings required for bath application of sevoflurane, which necessitated prolonged wash‐in and washout periods. These extended recording times often resulted in loss of seal resistance from the patch pipette, leading to the exclusion of some datasets. Additionally, when sevoflurane was combined with Tityustoxin‐Κα (TsTX‐Κα), we observed accelerated dechlorination of the bath electrode. This caused voltage drift and eventual loss of recordings, possibly due to ionic exchange between fluoride ions in the bath and Ag/AgCl electrodes. To mitigate this issue, we routinely re‐chlorinated electrodes between recordings involving both sevoflurane and TsTX‐Κα.

Another limitation was our inability to systematically quantify the latency to the first spike across conditions. This was due to the use of various ramp protocols, which were necessary to account for the substantial variability in action potential (AP) threshold following sevoflurane application—particularly in type A pyramidal neurons. Furthermore, our study does not directly reflect clinical concentrations of sevoflurane. First, the vaporizer used was not specifically calibrated for sevoflurane, and second, the anesthetic was applied via constant aqueous perfusion rather than through a gas phase, making it incomparable to alveolar absorption observed in vivo. Previous research has identified the minimum alveolar concentration (MAC) of sevoflurane to be approximately 2.4% in humans and 2.7% (~0.40 mM) in rats (Eger et al. 1965; Nishikawa and MacIver 2001). In our study, we found that saturated bath application of 22 μM sevoflurane (following 40 min of pre‐bubbling and > 15 min of application) was sufficient to reduce neuronal firing in slices of the auditory cortex of mice.

It may also be informative to investigate potential effects of sevoflurane on synaptic transmission. However, such experiments are technically demanding, as maintaining a stable aqueous concentration of this volatile anesthetic already makes recordings time‐consuming. Additional wash‐in and wash‐out steps required for agents such as TTX, CNQX, AP5, or picrotoxin would further increase the likelihood of pipette seal loss and deterioration in recording quality. Any potential modulation of these circuit‐level processes by sevoflurane lies beyond the scope of the present study. It is therefore important to acknowledge that some of the parameter adjustments emerging from our modeling procedure may represent compensation for unmodeled synaptic influences.

Despite these technical challenges, our findings show that low‐dose sevoflurane significantly reduces the firing frequency of L5 PNs and induces distinct, cell‐type‐specific changes in intrinsic membrane properties, categorized here as type A and type B neurons. In type A neurons, sevoflurane depolarized the resting membrane potential, whereas in type B neurons, it increased input resistance and altered action potential (AP) characteristics, including reduced amplitude and prolonged half‐width. These effects differ from those reported for anesthetics such as propofol, which typically hyperpolarize the resting membrane potential and decrease input resistance in both pyramidal neurons and fast‐spiking interneurons (Kaneko et al. 2016). Nevertheless, our measured inhibitory effects of sevoflurane on L5 PNs strongly suggest that sevoflurane, like other general anesthetics including isoflurane, ketamine/xylazine, and urethane, may also attenuate higher‐order feedback by decoupling distal dendritic input from somatic output in L5 PNs of the neocortex (Aru et al. 2020; Suzuki and Larkum 2020). This decoupling likely contributes to cortical disconnection and loss of conscious sensory experience. In line with this mechanism, sevoflurane is expected to promote sensory unawareness and isolation from the external environment (Kelz and Mashour 2019), potentially by enhancing synchronous oscillatory activity among cortical pyramidal neurons and interneurons (Bharioke et al. 2022), a compelling hypothesis that merits further investigation in vivo.

In our study, a physiologically relevant low concentration of sevoflurane was sufficient to reduce or abolish repetitive firing in response to depolarizing current injections in L5 pyramidal neurons. The partial reversal of these effects at subthreshold potentials (−50 mV) by TsTX‐Kα, a selective Kv1.2 channel blocker, implicates Kv1.2 potentiation as a contributing mechanism in the suppression of excitability. This interpretation is supported at the molecular level by prior studies using molecular dynamics simulations and heterologous expression systems, which have shown that sevoflurane (0.05–1 mM) enhances Kv1.2 conductance, especially at hyperpolarized potentials, by shifting the channel's activation curve leftward (Barber et al. 2012; Liang et al. 2015; Stock et al. 2017, 2018). Conversely, TsTX‐Kα blocks Kv1.2 channels via extracellular binding, likely by interacting with the selectivity filter, without altering membrane capacitance (Finol‐Urdaneta et al. 2010; Treptow et al. 2024). The physiological relevance of Kv1.2 modulation is further supported by studies showing that antibody‐mediated inhibition of Kv1.2 in the central‐medial thalamus can transiently restore consciousness in anesthetized animals (Alkire et al. 2008), and that genetic disruption of Kv1.2 renders Drosophila resistant to volatile anesthetics (Kelz and Mashour 2019).

Activation of low‐threshold voltage‐gated K+ currents through Kv1 channels, allowing for K+ efflux, is a rare effect of general anesthetics, which more often increase inward Cl‐ conductance through GABAergic receptors (Hao et al. 2020). However, NEURON simulations using a biophysically detailed model of thick‐tufted L5b pyramidal neurons corroborated our findings by revealing a statistically significant increase in persistent K^+^ conductance following sevoflurane exposure (Figure 7; Figure S6). Although the modeling results cannot be directly compared to the experimental data shown in Figure 6, given that the simulations are based on fixed ionic conductances, whereas Figure 6 reflects measurements at fixed voltages, they provide mechanistic insight into how sevoflurane reduces L5 PN excitability. Specifically, these findings support the idea that sevoflurane modulates Kv currents, including those active at subthreshold potentials, contributing to suppressed firing in both type A and type B neurons.

Beyond Kv1.2, sevoflurane is known to modulate other ion channel families, including tandem‐pore potassium (K2P) channels (Luethy et al. 2017), hyperpolarization‐activated cyclic nucleotide‐gated (HCN) channels (Nishikawa and MacIver 2000; Zhou et al. 2015; Schwerin et al. 2020), and voltage‐gated sodium (Nav) channels (Barber et al. 2014). This broad multitarget profile likely accounts for the partial reversal of anesthetic effects observed with TsTX‐Kα, which selectively blocks Kv1.2 channels.

In type A neurons, the experimentally observed changes in afterhyperpolarization amplitude and firing adaptation in the presence and absence of sevoflurane indicate functional involvement of SK channels in anesthetic action (Dreixler et al. 2000; Perry et al. 2015). Although further studies will be required to confirm this hypothesis, our NEURON simulations support a role for SK channels in mediating the effects of sevoflurane. The molecular mechanism by which sevoflurane enhances SK activity is unclear, as a direct effect of volatile general anesthetics on SK channels has not yet been demonstrated experimentally (Li et al. 2018). In this context, determining whether the SK channel blocker apamin, alone or in combination with calcium channel blockers or calcium store modulators, can counteract the membrane effects of sevoflurane in layer 5 pyramidal neurons represents an important avenue for future investigation.

In type B cells, sevoflurane depolarized the AP threshold, reduced AP amplitude, and significantly increased AP half‐width—findings suggesting altered Nav channel function. Supporting this interpretation, our simulations corresponding to type B neurons revealed a significant increase in persistent sodium conductance under sevoflurane, indicating that the interplay between low‐threshold Kv1.2 activation and enhanced Nav availability may contribute to the overall suppression of excitability in L5 pyramidal neurons. Additionally, sevoflurane induced larger hyperpolarization amplitudes in response to negative current injections, likely reflecting an increase in input resistance in type B cells. These results suggest that sevoflurane may suppress *I*_h_ in L5 pyramidal neurons—a hypothesis that warrants further investigation through both experimental and computational approaches.

## 5 Conclusions

To our knowledge, this is the first study to demonstrate distinct, cell‐type‐specific effects of sevoflurane on layer 5 pyramidal neurons (L5 PNs) and to identify Kv1.2 channel potentiation as a contributing mechanism. While previous studies have shown that sevoflurane uniquely stabilizes the open state of Kv1.2 potassium channels compared to other anesthetics (Barber et al. 2011; Covarrubias et al. 2015; Liang et al. 2015; Stock et al. 2018), our findings are the first to establish the functional relevance of this interaction in modulating L5 PN excitability—potentially elucidating the role of Kv1.2 channels in anesthetic‐induced outcomes (Alkire et al. 2008).

Taken together, these results offer novel insights into the molecular mechanisms of general anesthesia and are likely to be of broad interest to the neuroscience community. Given the significance and timeliness of these findings, we anticipate that the novel insights presented here might provide relevant information for future studies and should contribute to advancing our knowledge in the field.

## Author Contributions

**Aelton S. Araujo:** investigation, data curation, visualization, formal analysis, writing – original draft. **Gabriel M. de Queiroz:** data curation, formal analysis, investigation, writing – original draft. **Sérgio Ruschi B. Silva:** methodology, formal analysis, validation, writing – original draft. **Werner Treptow:** conceptualization, data curation, writing – review and editing, funding acquisition, supervision, project administration, resources. **Katarina E. Leao:** visualization, conceptualization, writing – review and editing, funding acquisition, supervision, project administration, resources.

## Funding

This work was supported by the FAPDF grant number 00193‐00001721/2024‐66, Coordination for the Improvement of Higher Education Personnel (CAPES), and the Brazilian National Council for Scientific and Technological Development (CNPq).

## Conflicts of Interest

The authors declare no conflicts of interest.

## Supporting information


**Table S1:** Average membrane and firing properties of L5 type A and type B.
**Table S2:** Full statistical report for Linear Mixed Model (LMM) analyses.
**Table S3:** Model comparison statistics from U‐Mann–Whitney test for Type.
**Table S4:** Comparison of electrophysiological parameters from patch clamp.
**Table S5:** Model comparison statistics from U‐Mann–Whitney test for Type.
**Figure S1:** Quantification of Sevoflurane using gas chromatography‐mass spectrometry.
**Figure S2:** Experimental aCSF data and model prediction.
**Figure S3:** Experimental sevo and sevo + TsTX data and model.
**Figure S4:** Model convergence history for aCSF condition of Type A.
**Figure S5:** Model convergence history for sevo and sevo + TsTX.
**Figure S6:** Comparison of experimental data with a Prediction model.

## Data Availability

All data considered in the study, including recorded data of L5PNs and scripts for Neuron simulations, can be downloaded from GIN repository https://gin.g‐node.org/aeltona/Sevoflurane‐L5.git.
