# Supplementary material for: Sevoflurane Inhibits Layer 5 Pyramidal Neurons via Kv1.2‐Dependent Modulation of Subthreshold Currents
Source: J Neurochem. 2026 Jan 20;170(1):e70360. doi: 10.1111/jnc.70360 (PMC12817328; doi:10.1111/jnc.70360)
Supplement: Supplementary file 1 — Table S1: Average membrane and firing properties of L5 type A and type B. Table S2: Full statistical report for Linear Mixed Model (LMM) analyses. Table S3: Model comparison statistics from U‐Mann–Whitney test for Type. Table S4: Comparison of electrophysiological parameters from patch clamp. Table S5: Model comparison statistics from U‐Mann–Whitney test for Type. Figure S1: Quantification of Sevoflurane using gas chromatography‐mass spectrometry. Figure S2: Experimental aCSF data and model prediction. Figure S3: Experimental sevo and sevo + TsTX data and model. Figure S4: Model convergence history for aCSF condition of Type A. Figure S5: Model convergence history for sevo and sevo + TsTX. Figure S6: Comparison of experimental data with a Prediction model. [file JNC-170-0-s001.pdf]

Supplementary Material

Sevoflurane Inhibits Layer 5 Pyramidal Neurons via Kv1.2-Dependent Modulation of Subthreshold Currents

Authors: Aelton S. Araújo[1], Gabriel M. de Queiroz[1], Sérgio Ruschi B. Silva[1], Werner Treptow[2*] and Katarina E. Leao[1*]

Author affiliation:

1. Hearing and Neuronal Activity lab, Brain Institute, Federal University of Rio Grande do Norte, RN 59078-900, Natal, Brazil
2. Laboratório de Biologia Teórica e Computacional, Departamento de Biologia Celular, Universidade de Brasília, DF 70910-900, Brasília, Brazil

Supplementary Table S1. Average membrane and firing properties of L5 type A and type B PNs.

	**t-statistic** degrees of freedom = 35	**Type A**	**Type B**	**All aCSF**
		**aCSF** n=21 **mean ± SEM**	**aCSF** n=16 **mean ± SEM**	**Type A vs Type B p-value**
Vm (mV)	1.64	-64.26 ± 1.24	-67.29 ± 1.35	0.22
Rin (MΩ)	1.69	196.85 ± 18.20	157.92 ± 11.10	0.22
AP Threshold (mV)	-1.52	-39.97 ± 0.99	-37.94 ± 0.81	0.22
Rheobase (pA)	-1.12	75.08 ± 7.66	86.76 ± 6.53	0.34
AP Half-width (ms)	0.85	1.71 ± 0.11	1.59 ± 0.07	0.40
AP Amplitude (mV)	-1.72	78.43 ± 1.77	82.69 ± 1.62	0.22
Δsag (mV)	5.52	3.56 ± 0.33	1.18 ± 0.23	*2.1e-5**
Hyperpol. (mV)	1.52	26.83 ± 2.60	21.8 ± 1.63	0.22
ΔADP (mV)	6.06	3.58 ± 0.33	0.99 ± 0.21	*1.2e-5**
ΔAHP (mV)	5.56	4.02 ± 0.43	1.12 ± 0.18	*2.1e-5**
FF at 50 pA (Hz)	1.75	3.62 ± 1.11	1.25 ± 0.5	0.22
FF at 150 pA (Hz)	1.38	14.24 ± 1.77	11.19 ± 0.97	0.24
FF at 250 pA (Hz)	0.91	18.19 ± 2.14	15.56 ± 1.76	0.39
FF at 350 pA (Hz)	1.02	21.24 ± 2.85	17.31 ± 2.32	0.37
FF at 450 pA (Hz)	1.49	21.00 ± 2.74	14.88 ± 3.04	0.22

Student's two-sided t-test with p-values after Benjamini-Hochberg false discovery rate correction. Mean ± S.E.M. * $p < 0.05$.

Supplementary Table S2. Full statistical report for Linear Mixed Model (LMM) analyses. The table presents the global effect of experimental treatment using Wald F-statistics, reported as $F_{(df\ between,\ df\ within)}$ with raw P-values, for type A and type B neurons. Subsequent columns detail the pairwise contrasts between conditions, including the model parameter estimate (beta) and the Wald z-score.

	Type A					Type B			
	Wald F-test	*β*	*z-score*	*p-value*	Comparison	*Wald F-test*	*β*	*z-score*	*p-value*
Vm (mV)	$F_{(2,35)} = 5.08$ p = 0.01	11.06	3.00	*0.006**	aCSF vs Sevo	$F_{(2,29)} = 1.95$ p = 0.16	6.91	1.86	0.11
		-4.35	-1.01	0.59	Sevo vs Sevo + TsTX		-1.05	-0.22	0.87
		6.71	1.81	0.12	aCSF vs Sevo + TsTx		5.86	1.20	0.33
Rin (MΩ)	$F_{(2,35)} = 2.50$ p = 0.10	-28.58	-1.93	0.10	aCSF vs Sevo	$F_{(2,29)} = 7.41$ p = 003	42.9	3.28	*0.002**
		0.14	0.01	0.99	Sevo vs Sevo + TsTX		-54.3	-2.82	*0.03**
		-28.06	-1.78	0.12	aCSF vs Sevo + TsTx		-12.67	-0.75	0.51
AP Thres. (mV)	$F_{(2,35)} = 10.8$ p = 2.3e-4	13.82	4.42	*3.1e-5**	aCSF vs Sevo	$F_{(2,29)} = 6.45$ p = 0.005	6.94	3.47	*0.001**
		-4.29	-1.23	0.59	Sevo vs Sevo + TsTX		-5.84	-2.15	0.12
		9.50	2.85	*0.01**	aCSF vs Sevo + TsTx		0.84	0.31	0.81
Rheobase (pA)	$F_{(2,35)} = 2.16$ p = 0.13	8.00	0.55	0.71	aCSF vs Sevo	$F_{(2,29)} = 6.65$ p = 0.004	-23.82	-2.73	*0.01**
		22.78	1.46	0.59	Sevo vs Sevo + TsTX		36.47	3.10	*0.02**
		32.75	2.07	0.07	aCSF vs Sevo + TsTx		12.96	1.11	0.35
AP Amp. (mV)	$F_{(2,35)} = 6.84$ p = 0.003	-7.52	-3.69	*0.001**	aCSF vs Sevo	$F_{(2,29)} = 7.26$ p = 0.003	-9.73	-3.76	*4.8e-4**
		5.13	2.19	0.54	Sevo vs Sevo + TsTX		3.81	1.06	0.42
		-2.71	-1.25	0.29	aCSF vs Sevo + TsTx		-5.49	-1.67-	0.16
AP HW (ms)	$F_{(2,35)} = 4.46$ p = 0.02	0.29	1.88	0.10	aCSF vs Sevo	$F_{(2,29)} = 4.82$ p = 0.01	0.41	2.79	*0.01**
		0.15	0.90	0.59	Sevo vs Sevo + TsTX		0.004	0.02	0.99
		0.46	2.83	*0.01**	aCSF vs Sevo + TsTx		0.40	2.09	0.07
Δsag (mV)	$F_{(2,35)} = 2.74$ p = 0.08	-0.64	-1.30	0.27	aCSF vs Sevo	$F_{(2,29)} = 4.74$ p = 0.02	1.22	2.68	*0.02**
		-0.54	-0.93	0.59	Sevo vs Sevo + TsTX		-1.51	-2.66	*0.04**
		-1.20	-2.31	*0.04**	aCSF vs Sevo + TsTx		-0.33	-0.53	0.65

Hyper pol. (mV)	$F_{(2,35)}$ = 4.53 p = 0.02	8.01	2.91	0.008*	aCSF vs Sevo	$F_{(2,29)}$ = 13.26 p = 8.1e-5	10.98	5.07	1.4e-6*
		-8.30	-1.85	0.59	Sevo vs Sevo + TsTX		-3.7	-1.37	0.27
		1.18	0.40	0.80	aCSF vs Sevo + TsTx		6.89	2.59	0.02*
ΔADP (mV)	$F_{(2,35)}$ = 1.56 p = 0.23	-0.85	-1.21	0.30	aCSF vs Sevo	$F_{(2,29)}$ = 2.54 p = 0.10	0.92	2.16	0.06
		-0.36	-0.47	0.76	Sevo vs Sevo + TsTX		-0.88	-1.58	0.22
		-1.19	-1.63	0.16	aCSF vs Sevo + TsTx		0.07	0.12	0.94
ΔAHP (mV)	$F_{(2,35)}$ = 0.74 p = 0.49	0.14	0.17	0.96	aCSF vs Sevo	$F_{(2,29)}$ = 7.81 p = 0.002	0.30	0.71	0.52
		-1.17	-1.17	0.59	Sevo vs Sevo + TsTX		1.81	3.19	0.02*
		-0.99	-1.10	0.34	aCSF vs Sevo + TsTx		2.13	3.94	2.4e-4*
FF at 50 pA (Hz)	$F_{(2,35)}$ = 0.61 p = 0.55	-1.79	-1.10	0.35	aCSF vs Sevo	$F_{(2,29)}$ = 0.49 p = 0.61	-0.03	-0.06	0.98
		1.62	0.92	0.59	Sevo vs Sevo + TsTX		-0.69	-0.98	0.44
		-0.42	-0.21	0.93	aCSF vs Sevo + TsTx		-0.69	-0.96	0.41
FF at 150 pA (Hz)	$F_{(2,35)}$ = 7.95 p = 0.001	-9.54	-3.45	0.001*	aCSF vs Sevo	$F_{(2,29)}$ = 16.78 p = 1.4e-5	-7.12	-5.40	2.6e-7*
		1.78	0.58	0.72	Sevo vs Sevo + TsTX		0.89	0.47	0.71
		-8.51	-2.86	0.01*	aCSF vs Sevo + TsTx		-6.33	-3.70	5.7e-4*
FF at 250 pA (Hz)	$F_{(2,35)}$ = 11.1 p = 1.8e-4	-14.41	-4.25	6.1e-5*	aCSF vs Sevo	$F_{(2,29)}$ = 22.48 p = 1.3e-6	-10.75	-6.49	4.2e-10 *
		4.22	1.06	0.59	Sevo vs Sevo + TsTX		3.38	1.49	0.24
		-10.44	-3.00	0.01*	aCSF vs Sevo + TsTx		-7.67	-3.75	4.8e-4*
FF at 350 pA (Hz)	$F_{(2,35)}$ = 10.98 p = 2.0e-4	-18.46	-4.37	3.8e-5*	aCSF vs Sevo	$F_{(2,29)}$ = 17.28 p = 1.1e-5	-13.89	-5.84	2.3e-8*
		5.72	1.14	0.59	Sevo vs Sevo + TsTX		6.17	2.04	0.13
		-12.74	-2.88	0.01*	aCSF vs Sevo + TsTx		-7.86	-2.61	0.02*

FF at 450 pA (Hz)	$F_{(2,35)}$ = 19.15 p = 2.0e-6	-15.99	-5.35	3.4e-7*	aCSF vs Sevo	$F_{(2,29)}$ = 8.40 p = 0.001	-11.93	-4.09	1.3e-4*
		0.56	0.17	0.91	Sevo vs Sevo + TsTX		5.90	1.56	0.22
		-14.99	-4.92	3e-6*	aCSF vs Sevo + TsTx		-5.57	-1.50	0.21
-77 mV bin	$F_{(2,14)}$ = 0.52 p = 0.61	-29.10	-1.00	0.52	aCSF vs Sevo	$F_{(2,11)}$ = 3.24 p = 0.08	-5.75	-0.58	0.70
		10.52	0.39	0.76	Sevo vs Sevo + TsTX		-16.34	-1.64	0.36
		-18.58	-0.69	0.68	aCSF vs Sevo + TsTx		-22.10	-2.47	0.21
-72 mV bin	$F_{(2,14)}$ = 0.72 p = 0.50	-34.32	-1.20	0.44	aCSF vs Sevo	$F_{(2,11)}$ = 2.23 p = 0.15	-13.74	-1.40	0.37
		19.25	0.72	0.68	Sevo vs Sevo + TsTX		-6.05	-0.62	0.70
		-15.07	-0.59	0.70	aCSF vs Sevo + TsTx		-19.79	-2.06	0.30
-67 mV bin	$F_{(2,14)}$ = 0.99 p = 0.39	-42.47	-1.39	0.37	aCSF vs Sevo	$F_{(2,11)}$ = 2.14 p = 0.16	-19.17	-1.70	0.31
		29.97	1.06	0.50	Sevo vs Sevo + TsTX		1.00	0.09	0.93
		-12.49	-0.49	0.75	aCSF vs Sevo + TsTx		-18.17	-1.78	0.31
-62 mV bin	$F_{(2,14)}$ = 1.15 p = 0.34	-49.88	-1.47	0.37	aCSF vs Sevo	$F_{(2,11)}$ = 1.82 p = 0.21	-22.69	-1.87	0.31
		39.35	1.28	0.42	Sevo vs Sevo + TsTX		8.55	0.70	0.68
		-10.54	-0.40	0.76	aCSF vs Sevo + TsTx		-14.14	-1.23	0.43
-57 mV bin	$F_{(2,14)}$ = 1.22 p = 0.32	-55.85	-1.44	0.37	aCSF vs Sevo	$F_{(2,11)}$ = 1.69 p = 0.23	-25.01	-1.84	0.31
		49.94	1.46	0.37	Sevo vs Sevo + TsTX		15.06	1.11	0.48
		-5.90	-0.21	0.88	aCSF vs Sevo + TsTx		-9.95	-0.78	0.68
-52 mV bin	$F_{(2,14)}$ = 1.23 p = 0.32	-29.18	-1.40	0.37	aCSF vs Sevo	$F_{(2,11)}$ = 1.82 p = 0.21	-28.41	-2.55	0.21
		36.47	2.04	0.30	Sevo vs Sevo + TsTX		26.46	2.37	0.21
		7.29	0.41	0.76	aCSF vs Sevo + TsTx		-1.95	-0.18	0.88
-77 to -62 mV slope	$F_{(2,14)}$ = 1.75 p = 0.21	-0.53	-0.62	0.58	aCSF vs Sevo	$F_{(2,11)}$ = 7.38 p = 0.009	-1.00	-2.50	0.05
		1.31	1.79	0.18	Sevo vs Sevo + TsTX		1.53	3.82	0.002
		0.78	1.09	0.37	aCSF vs Sevo + TsTx		0.53	1.42	0.31
-62 to -47 mV slope	$F_{(2,14)}$ = 1.03 p = 0.38	-0.03	-0.03	0.98	aCSF vs Sevo	$F_{(2,11)}$ = 3.57 p = 0.06	-0.48	-0.92	0.43
		0.93	1.15	0.37	Sevo vs Sevo + TsTX		1.35	2.59	0.05
		0.90	1.15	0.37	aCSF vs Sevo + TsTx		0.87	1.79	0.18

Supplementary Table S3. Model comparison statistics from U-Mann-Whitney test for Type A layer 5 pyramidal neuron.

ID	Comparison	Parameter	U-Statistic	p-Value	Significant
1	aCSF vs Sevo	gNaTa_tbar_NaTa_t	79.0	0.497237	False
2	aCSF vs Sevo	gNap_Et2bar_Nap_Et2	81.0	0.556310	False
3	aCSF vs Sevo	gK_Tstbar_K_Tst	69.0	0.257884	False
4	aCSF vs Sevo	gK_Pstbar_K_Pst	25.0	0.001771	**True**
5	aCSF vs Sevo	gSKv3_1bar_SKv3_1	105.0	0.650864	False
6	aCSF vs Sevo	gSK_E2bar_SK_E2	37.0	0.009858	**True**
7	aCSF vs Sevo	gCa_HVAbar_Ca_HVA	93.0	0.963903	False
8	aCSF vs Sevo	gCa_LVAstbar_Ca_LVAst	76.0	0.415294	False
9	aCSF vs Sevo	decay_CaDynamics_E2	92.0	0.927848	False
10	aCSF vs Sevo	gamma_CaDynamics_E2	102.0	0.746867	False
11	aCSF vs Sevo+TsTX	gNaTa_tbar_NaTa_t	50.0	0.102979	False
12	aCSF vs Sevo+TsTX	gNap_Et2bar_Nap_Et2	78.0	0.792660	False
13	aCSF vs Sevo+TsTX	gK_Tstbar_K_Tst	95.0	0.615341	False
14	aCSF vs Sevo+TsTX	gK_Pstbar_K_Pst	47.0	0.074551	False
15	aCSF vs Sevo+TsTX	gSKv3_1bar_SKv3_1	59.0	0.237274	False
16	aCSF vs Sevo+TsTX	gSK_E2bar_SK_E2	35.0	0.017898	**True**
17	aCSF vs Sevo+TsTX	gCa_HVAbar_Ca_HVA	76.0	0.719902	False
18	aCSF vs Sevo+TsTX	gCa_LVAstbar_Ca_LVAst	82.0	0.942877	False

19	aCSF vs Sevo+TsTX	decay_CaDynamics_E2	51.0	0.114091	False
20	aCSF vs Sevo+TsTX	gamma_CaDynamics_E2	91.0	0.746078	False
21	Sevo vs Sevo+TsTX	gNaTa_tbar_NaTa_t	29.0	0.541423	False
22	Sevo vs Sevo+TsTX	gNap_Et2bar_Nap_Et2	35.0	0.962567	False
23	Sevo vs Sevo+TsTX	gK_Tstbar_K_Tst	52.0	0.138791	False
24	Sevo vs Sevo+TsTX	gK_Pstbar_K_Pst	49.0	0.235870	False
25	Sevo vs Sevo+TsTX	gSKv3_1bar_SKv3_1	23.0	0.235870	False
26	Sevo vs Sevo+TsTX	gSK_E2bar_SK_E2	30.0	0.605841	False
27	Sevo vs Sevo+TsTX	gCa_HVAbar_Ca_HVA	35.0	0.962567	False
28	Sevo vs Sevo+TsTX	gCa_LVAstbar_Ca_LVAst	42.0	0.605841	False
29	Sevo vs Sevo+TsTX	decay_CaDynamics_E2	24.0	0.268179	False
30	Sevo vs Sevo+TsTX	gamma_CaDynamics_E2	37.0	0.959486	False

Supplementary Table S4. Comparison of electrophysiological parameters from patch clamp recordings and NEURON simulations of type A PN membrane properties (corrected for junction potential). The electrophysiological parameters were computed based on the experimental and simulated traces shown in Supplementary Figures S2 - S3.

	**aCSF**		**Sevo**		**Sevo + TsTX**	
	Experimental	Simulated	Experimental	Simulated	Experimental	Simulated
**RMP Mean ± SD**	-78.23 ± 6.09	-86.96 ± 0.16	-72.48 ± 11.61	-86.97 ± 0.17	-69.50 ± 11.05	-86.87 ± 0.08
**Mean Spike Count ± SD**	8.29 ± 4.40	7.19 ± 2.91	3.67 ± 2.96	3.33 ± 2.83	4.88 ± 4.64	4.00 ± 2.88
**AP Amplitude Mean ± SD**	25.89 ± 4.91	38.02 ± 7.21	22.91 ± 5.17	41.37 ± 8.60	34.10 ± 6.73	39.75 ± 6.20
**AP Threshold Mean ± SD**	-48.55 ± 8.56	-53.62 ± 2.13	-40.20 ± 17.64	-51.57 ± 2.47	-41.42 ± 9.09	-53.82 ± 2.45

RMP - resting membrane potential; spike count; AP - action potential amplitude; AP -action potential threshold; SD - standard deviation;

Supplementary Table S5. Model comparison statistics from U-Mann-Whitney test for Type B layer 5 pyramidal neuron.

ID	Comparison	Parameter	U-Statistic	p-Value	Significant
1	aCSF vs Sevo	gNaTa_tbar_NaTa_t	81.0	0.500855	False
2	aCSF vs Sevo	gNap_Et2bar_Nap_Et2	53.0	0.048494	**True**
3	aCSF vs Sevo	gK_Tstbar_K_Tst	114.0	0.416552	False
4	aCSF vs Sevo	gK_Pstbar_K_Pst	43.0	0.014799	**True**
5	aCSF vs Sevo	gSKv3_1bar_SKv3_1	57.0	0.073886	False
6	aCSF vs Sevo	gSK_E2bar_SK_E2	83.0	0.561714	False
7	aCSF vs Sevo	gCa_HVAbar_Ca_HVA	66.0	0.170842	False
8	aCSF vs Sevo	gCa_LVAstbar_Ca_LVAst	101.0	0.834521	False
9	aCSF vs Sevo	decay_CaDynamics_E2	60.0	0.099343	False
10	aCSF vs Sevo	gamma_CaDynamics_E2	117.0	0.340991	False
11	aCSF vs Sevo+TsTX	gNaTa_tbar_NaTa_t	45.0	0.718954	False
12	aCSF vs Sevo+TsTX	gNap_Et2bar_Nap_Et2	26.0	0.274805	False
13	aCSF vs Sevo+TsTX	gK_Tstbar_K_Tst	37.0	0.841712	False
14	aCSF vs Sevo+TsTX	gK_Pstbar_K_Pst	22.0	0.153030	False
15	aCSF vs Sevo+TsTX	gSKv3_1bar_SKv3_1	42.0	0.904713	False
16	aCSF vs Sevo+TsTX	gSK_E2bar_SK_E2	27.0	0.312743	**False**
17	aCSF vs Sevo+TsTX	gCa_HVAbar_Ca_HVA	59.0	0.129736	False
18	aCSF vs Sevo+TsTX	gCa_LVAstbar_Ca_LVAst	36.0	0.779793	False
19	aCSF vs Sevo+TsTX	decay_CaDynamics_E2	20.0	0.109195	False

20	aCSF vs Sevo+TsTX	gamma_CaDynamics_E2	77.0	0.002571	**True**
21	Sevo vs Sevo+TsTX	gNaTa_tbar_NaTa_t	41.0	0.278604	False
22	Sevo vs Sevo+TsTX	gNap_Et2bar_Nap_Et2	30.0	1.000000	False
23	Sevo vs Sevo+TsTX	gK_Tstbar_K_Tst	26.0	0.721396	False
24	Sevo vs Sevo+TsTX	gK_Pstbar_K_Pst	32.0	0.878798	False
25	Sevo vs Sevo+TsTX	gSKv3_1bar_SKv3_1	41.0	0.278604	False
26	Sevo vs Sevo+TsTX	gSK_E2bar_SK_E2	23.0	0.505818	False
27	Sevo vs Sevo+TsTX	gCa_HVAbar_Ca_HVA	51.0	0.026826	**True**
28	Sevo vs Sevo+TsTX	gCa_LVAstbar_Ca_LVAst	30.0	1.000000	False
29	Sevo vs Sevo+TsTX	decay_CaDynamics_E2	31.0	0.959276	False
30	Sevo vs Sevo+TsTX	gamma_CaDynamics_E2	38.0	0.423456	False

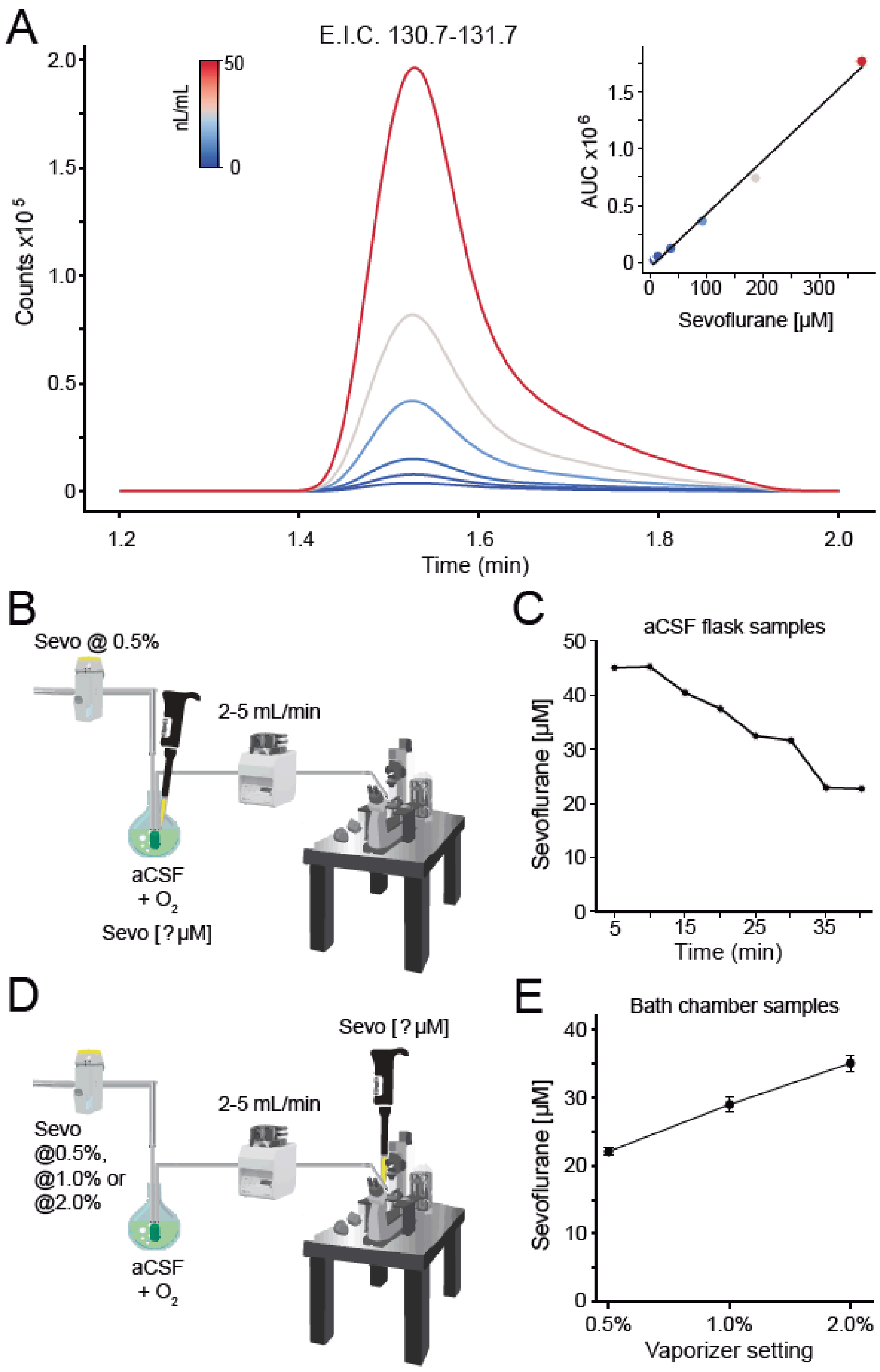

Supplementary Figure S1

Supplementary Figure S1. Quantification of Sevoflurane using gas chromatography-mass spectrometry**. A)** Calibration curves were acquired from samples of standardized serial dilutions. **B)** The vaporizer was loaded with sevoflurane (1mL/mL, 100% sevoflurane) and adjusted to 0.5% of pressurized carbogen (95% O_2, 5% CO_2) flow. At different time points (5-40 min, 5 min interval), samples of 1mL of aCSF were collected into gas tight vials from the aCSF flask constantly bubbled with carbogen. Sevoflurane concentration in the aCSF was extrapolated from the standard curve. **C)** Samples collected from the aCSF flask or from the recording chamber were comparable (22μM) when using the same vaporizer settings (0.5%) due to usage of gas-tight PTFE tubing for delivering the aCSF to the bath chamber. The concentration of sevoflurane in the recording chamber could be slightly increased by adjusting the vaporizer to a higher percentage (1%; 28μM, 2%; 35μM).

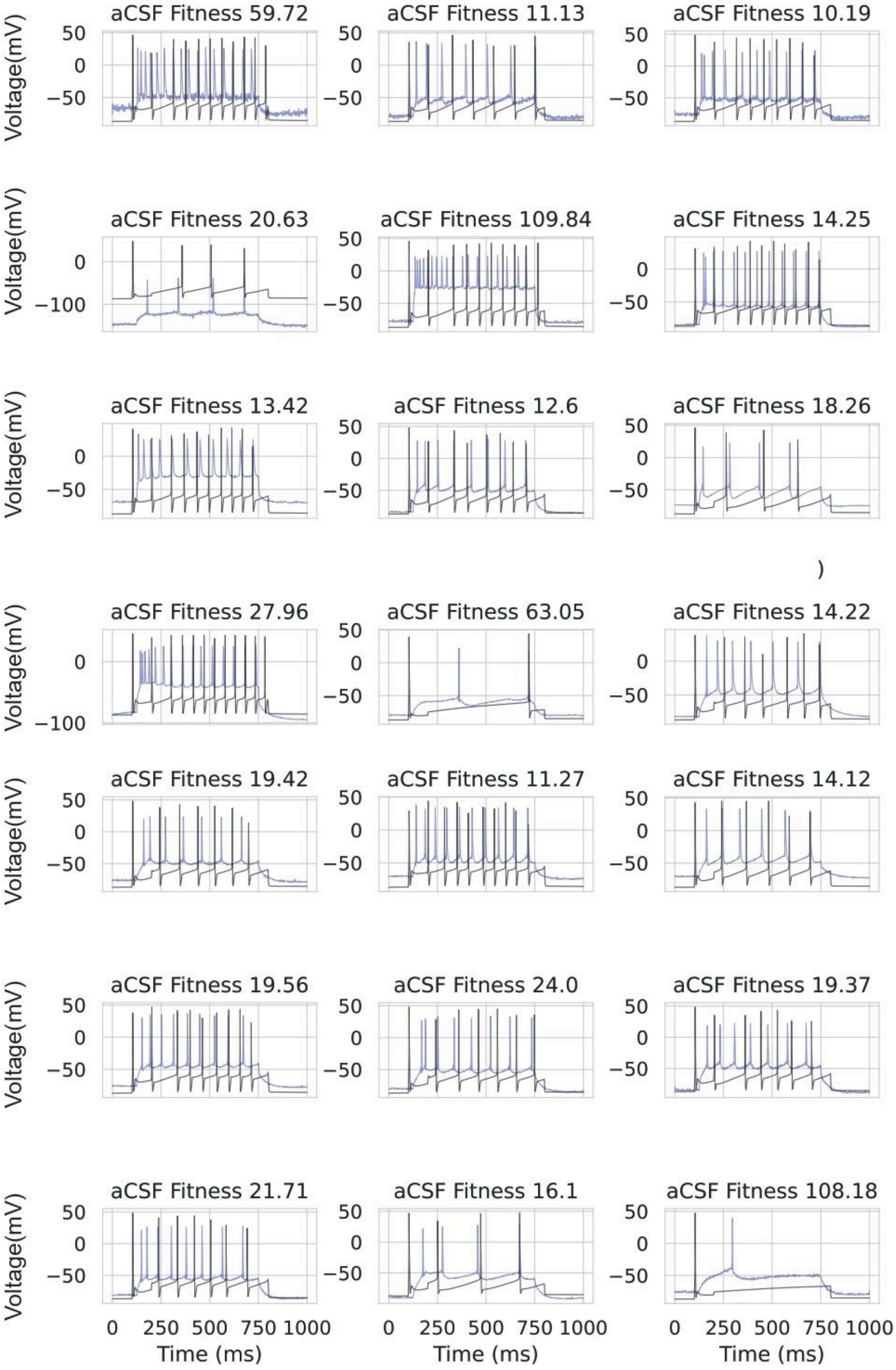

Supplementary Figure S2

Supplementary Figure S2. Experimental aCSF data and model prediction superposition for Type A PNs. Superposition of experimental traces (blue) and model prediction (black) for each individual neuron (n = 21) with best numerical fitness for each experimental trace. A lower number indicates better fitness of the model.

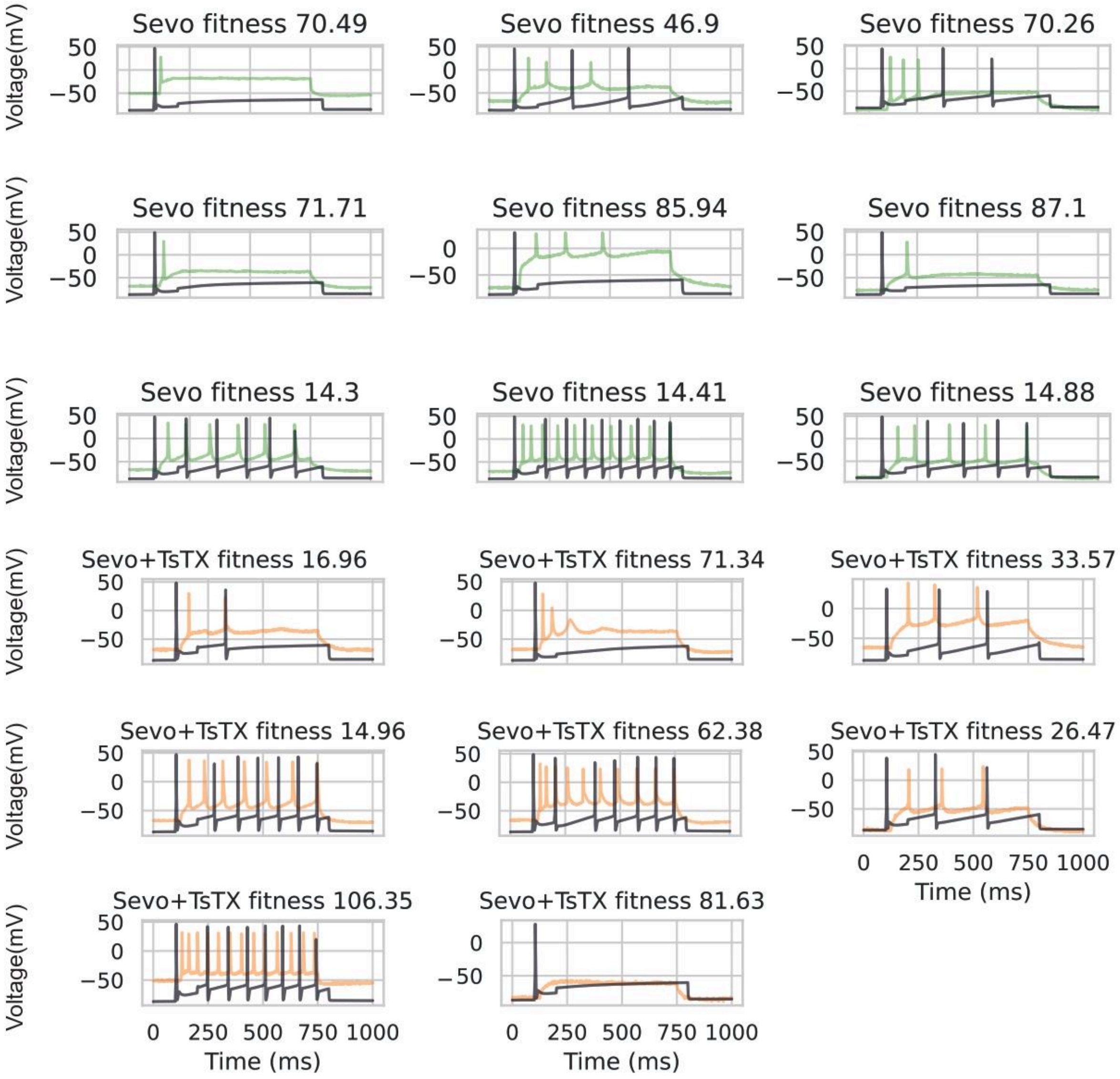

Supplementary Figure S3

Supplementary Figure S3. Experimental sevo and sevo +TsTX data and model prediction superposition for Type A PNs. Superposition of experimental traces for sevoflurane (green, n = 9) and sevo + TsTX (orange, n = 8) with best numerical fitness for each experimental trace. A lower number indicates better fitness of the model.

Supplementary Figure S4

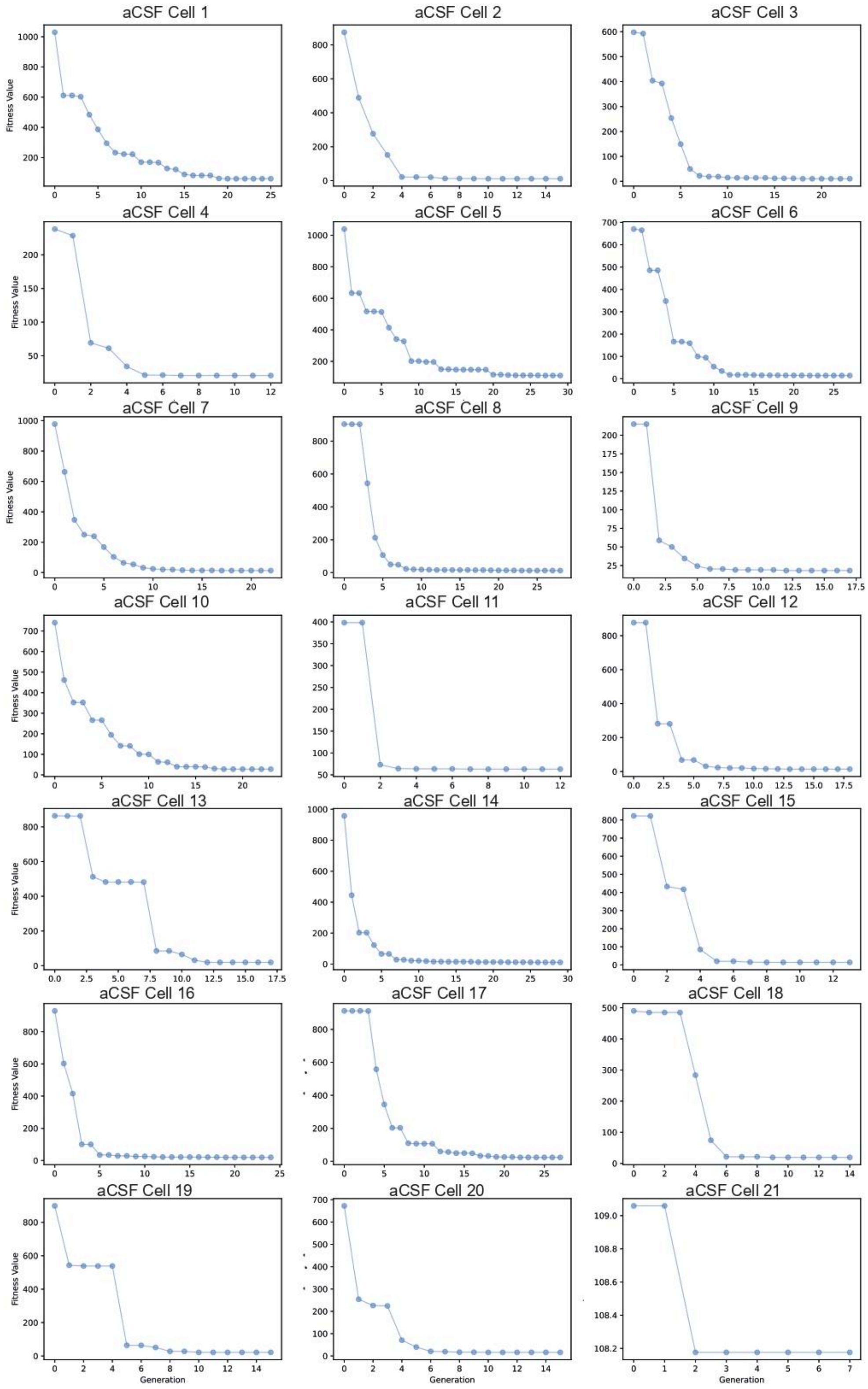

Supplementary Figure S4. Model convergence history for aCSF condition of Type A PNs. Convergence of the genetic algorithm used to fit experimental data with the neuronal model, each panel is an individual fitness history (n = 21). The blue trace shows the decrease in fitness value as the algorithm progresses through successive generations, this behavior displays the convergence to an optimal set of parameters.

Supplementary Figure S5

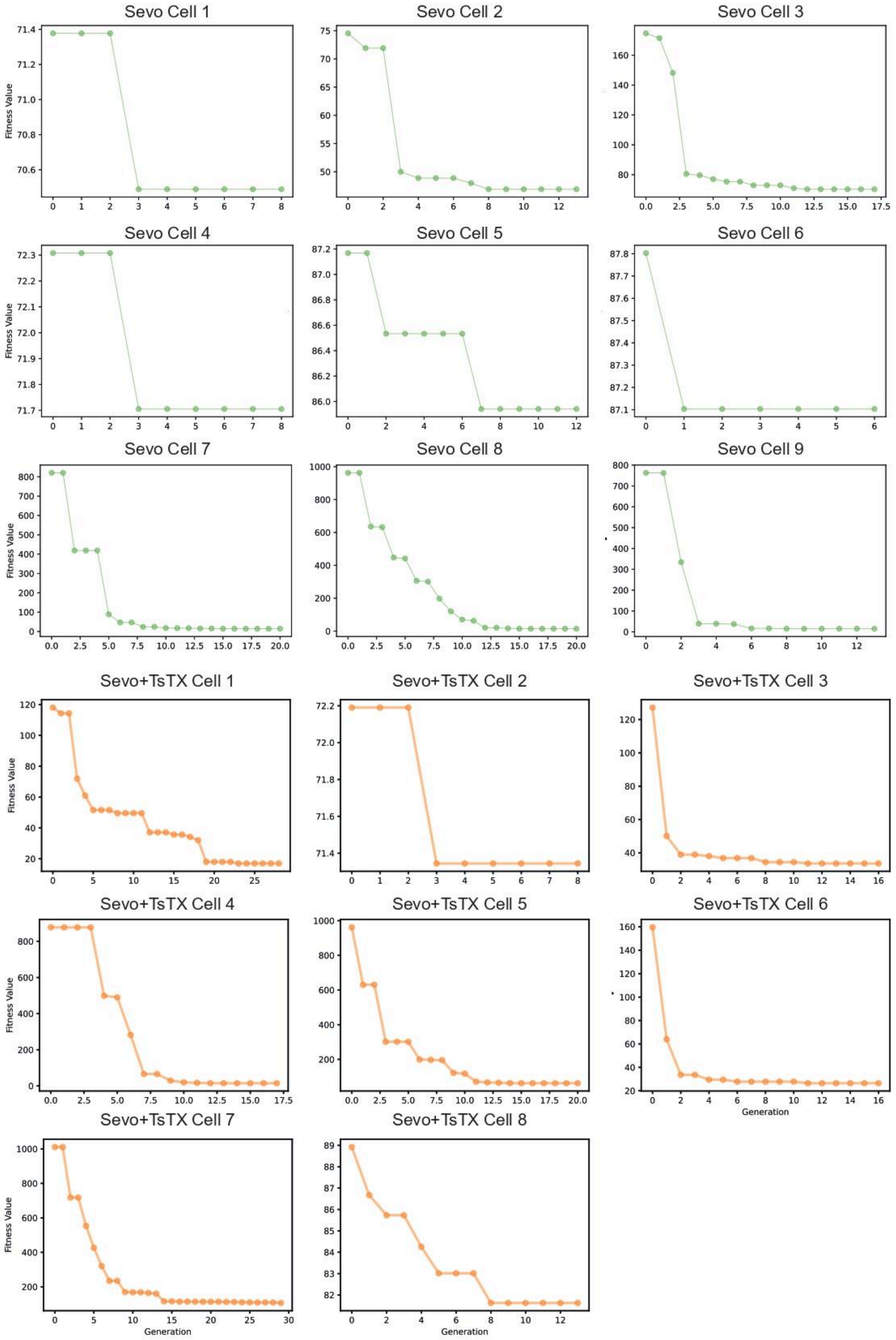

Supplementary Figure S5. Model convergence history for sevo and sevo + TsTX conditions of Type A PNs. Convergence of the genetic algorithm used to fit experimental data with the neuronal model, each panel is an individual fitness history for each cell in sevo sevoflurane (green, n = 9) and sevo + TsTX (orange, n = 8) , respectively. Graphs show the decrease in fitness value as the algorithm progresses through successive generations, displaying the convergence to an optimal set of parameters.

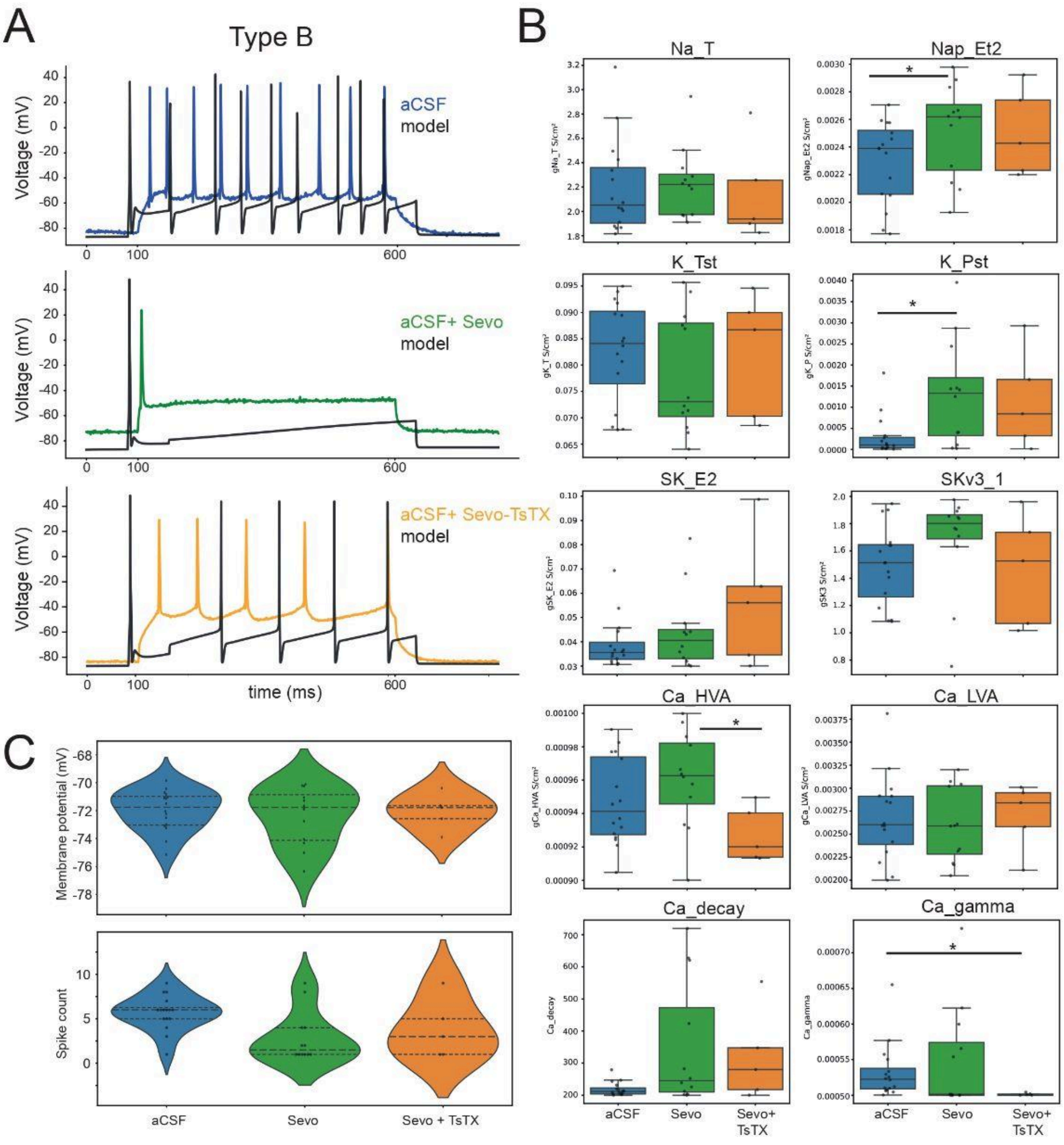

Supplementary Figure S6

Supplementary Figure S6. Comparison of experimental data with a Prediction model for type B PNs. *Top:* Overlay of experimentally recorded baseline neuronal trace (blue) and model-predicted trace (black) using estimated channel conductances. *Middle:* Experimental Sevoflurane traces (green) vs. modeled (black) traces after sevoflurane application. *Bottom:* Experimental (yellow) vs. modeled (black) traces under sevoflurane + Tityustoxin-Kα (Sevo+TsTX-Kα) conditions. **B)** Normalized conductance changes (median +- IQR) for Fast Na's (Na_T), Persistent Na's (Nap_Et2), Fast Kv's (K_Tst), Persistent Kv's (K_Pst), delayed rectifier K^+ (SKv3_1), calcium-activated potassium channels (SK_E2); high-voltage activated Ca^{2+} channels (Ca_HVA), low-voltage activated Ca2+ channels (Ca_LVA), and calcium delay rate and intracellular calcium buffering (gamma). aCSF n=16, Sevo n=12, Sevo+TsTX n =5. Boxplots show median (Q2) (horizontal bar), the box it's IQR, Whiskers 1.5 times IQR,full statistical report on Supplementary Table S4, *$p < 0.05$. **C)** Violin plots show distribution for simulated membrane potential and firing (spike count).
